# Biomaterial-mediated blood clot formation: a key to unlocking osteogenic synergy for bone regeneration

**DOI:** 10.1038/s41413-026-00559-9

**Published:** 2026-09-24

**Authors:** Dong Zhang, Laiqiang Tong, Lijie Mao, Dong Han, Fangping Chen, Changsheng Liu

**Affiliations:** 1 Engineering Research Center for Biomedical Materials of Ministry of Education, East China University of Science and Technology, Shanghai, PR China; 2 Department of Plastic and Reconstructive Surgery, Shanghai Jiao Tong University School of Medicine, Shanghai Ninth People’s Hospital, Shanghai, PR China; 3 Key Laboratory for Ultrafine Materials of Ministry of Education, School of Materials Science and Engineering, East China University of Science and Technology, Shanghai, PR China

**Keywords:** Bone, Pathogenesis

## Abstract

Bone hemorrhage presents serious clinical challenges due to its high morbidity and mortality. Autologous coagulation forms blood clots that both halt bleeding and provide a natural scaffold for bone repair. The dynamic formation and dissolution of blood clots maintain a delicate balance between hemostasis and regeneration. Clot architecture and cytokine composition play pivotal roles in directing bone repair. These insights inspire biomimetic strategies using engineered biomaterials to regulate clot behavior, a central theme in designing bone-replacement systems that integrate hemostasis with osteogenesis. This review highlights the processes of clot formation following bone trauma and the contribution of cytokine-mediated signaling to bone repair. Particular emphasis is placed on how biomaterial implants modulate clot function via their physicochemical properties, such as chemical composition, structural characteristics, hydrophilicity, and surface charge. Additionally, the review examines the role of the clots during the early stages of bone injury and discusses the rational design principles for materials aimed at optimizing coagulation control and osteogenesis. Overall, this review reframes the clot not as a passive by-product of trauma but as an interface through which biomaterials seamlessly merge hemostasis and osteogenesis, offering a roadmap for multifunctional bone-replacement systems that halt bleeding while programming robust bone repair.

## Introduction

Severe trauma, accounting for approximately 10% of the 58 million annual global deaths,^1^ has become a significant public health concern. Trauma-related mortality is frequently due to traffic accidents, battlefield trauma and postoperative complications.^2^ Around 40% of trauma-related deaths stem from uncontrollable bleeding, with bone trauma bleeding posing unique management challenges. For example, the 40% failure rate of spinal fusion bone grafts exemplifies the difficulties in managing bone injuries and achieving effective hemostasis,^3^ which is ascribed to the insufficient hemostasis of the scaffold and the suboptimal structural properties of the blood clot.^4^

The Haversian intraosseous vascular network makes hemorrhage after skeletal trauma exceptionally difficult to control, as these longitudinal vascular channels are embedded within rigid mineralized bone and lack the capacity for external compression, thereby sustaining persistent deep bleeding.^5^ Nevertheless, injured bones exhibit a remarkable capacity for repair, driven by four continuous, overlapping phases: clot formation, inflammation, bone formation, and remodeling.^6^ Within minutes of injury, blood from destroyed bone vessels fills the defect, forming a clot that is indispensable for initiating bone healing. The clot is primarily composed of erythrocytes (95%), platelets (<5%), and leukocytes (<1%).^7^ Platelets drive coagulation and osteogenesis by releasing proangiogenic and osteogenic growth factors^7^. Erythrocytes enhance platelet reactivity and improve fibrin network structures. Leukocytes contribute to immune responses by eliminating pathogens, secreting cytokines, and aiding extracellular matrix synthesis.^8^ In addition to these cellular interactions, bone traumatic blood clots recruit skeletal stem cells (SSCs), which exhibit characteristics similar to bone marrow-derived SSCs. These cells are recruited by inflammatory cytokines and growth factors from the periosteum and surrounding soft tissues during the initial 24 h post-bone trauma.^9^ As fibrin forms, the clots act as temporary reservoirs for various factors that guide the subsequent inflammatory response and osteogenesis. Thus, the formation and integrity of the fibrin network in clots play an important role in bone traumatic repair.^10,11^

Despite the fundamental role of blood clots in initiating bone repair, their therapeutic reliability is often compromised by several critical limitations.^12^ First, there is a distinct lack of precision in regulating clot architecture; native fibrin networks frequently exhibit suboptimal and uncontrollable structural conformations, which may lack the mechanical stability or the specific porosity required to support robust cell migration.^13^ Second, the efficacy of the growth factor reservoir within the clot is hampered by unpredictable and transient release profiles, which often fail to maintain the sustained signaling necessary for prolonged osteogenesis.^14^ These deficiencies culminate in a significant efficiency bottleneck for treating critical-sized bone defects.^15^ In such large-scale injuries, the spontaneous formation of a native blood clot is typically insufficient to bridge the gap or provide a stable enough scaffold, leading to a failure of spontaneous healing and high non-union rates. Consequently, there is an urgent need for biomaterial-mediated strategies that can actively program clot behavior to overcome these physiological limits. Recent studies have highlighted the significant influence of the interaction between blood clots and biomaterials on the healing process. This interaction is primarily governed by the physical, chemical, and mechanical properties of the biomaterials. Specifically, the material properties can modulate the behavior of the blood clot formed during hemostasis, thereby impacting cellular interactions and the healing time. Consequently, the synergistic modulation of blood clot formation by biomaterials offers a promising strategy to enhance the efficiency of bone repair.

Here, we review the molecular and ultrastructural events that govern clot formation in the early stages of bone traumatic and examine how clot structure and its cytokines jointly regulate hemostasis and subsequent osteoregeneration. We frame the early clot as a dynamic, bioactive microenvironment that integrates hemostasis and osteogenesis. In addition, particular attention is paid to the regulatory effects of biomaterials on clots, which significantly alter their structure and function. This article systematically classifies and summarizes recent studies on the mechanisms by which biomaterials mediate the clot formation, and provides an in-depth discussion of the biological effects arising from interactions between blood components and biomaterials during bone repair. This study provides insight into the design of implants that actively utilize the traumatic clot to achieve hemostasis control while accelerating orthotopic bone regeneration under the bone hemorrhagic conditions.

## Blood clot formation and mechanisms

### Blood clot formation and hemostasis

Coagulation at the site of bone trauma serves as the initial step in bone repair, thus maintaining homeostasis and avoiding necrosis at the bone defect ends caused by insufficient oxygen and nutrient supply.^16^ The blood clot forms a crucial connection and stabilization between the bone trauma bone fragments and the vascular injury, which is essential for coordinating an immediate response and promoting tissue repair.^17^ This process involves the transformation of an unstable platelet aggregate into a stable fibrin network. The fibrin network provides mechanical anchorage and a microenvironment for cellular adhesion and function.^18^ Recent studies have demonstrated that the structural properties of blood clots at bone trauma sites can influence bone regeneration.^19^

**Fig. 1:**
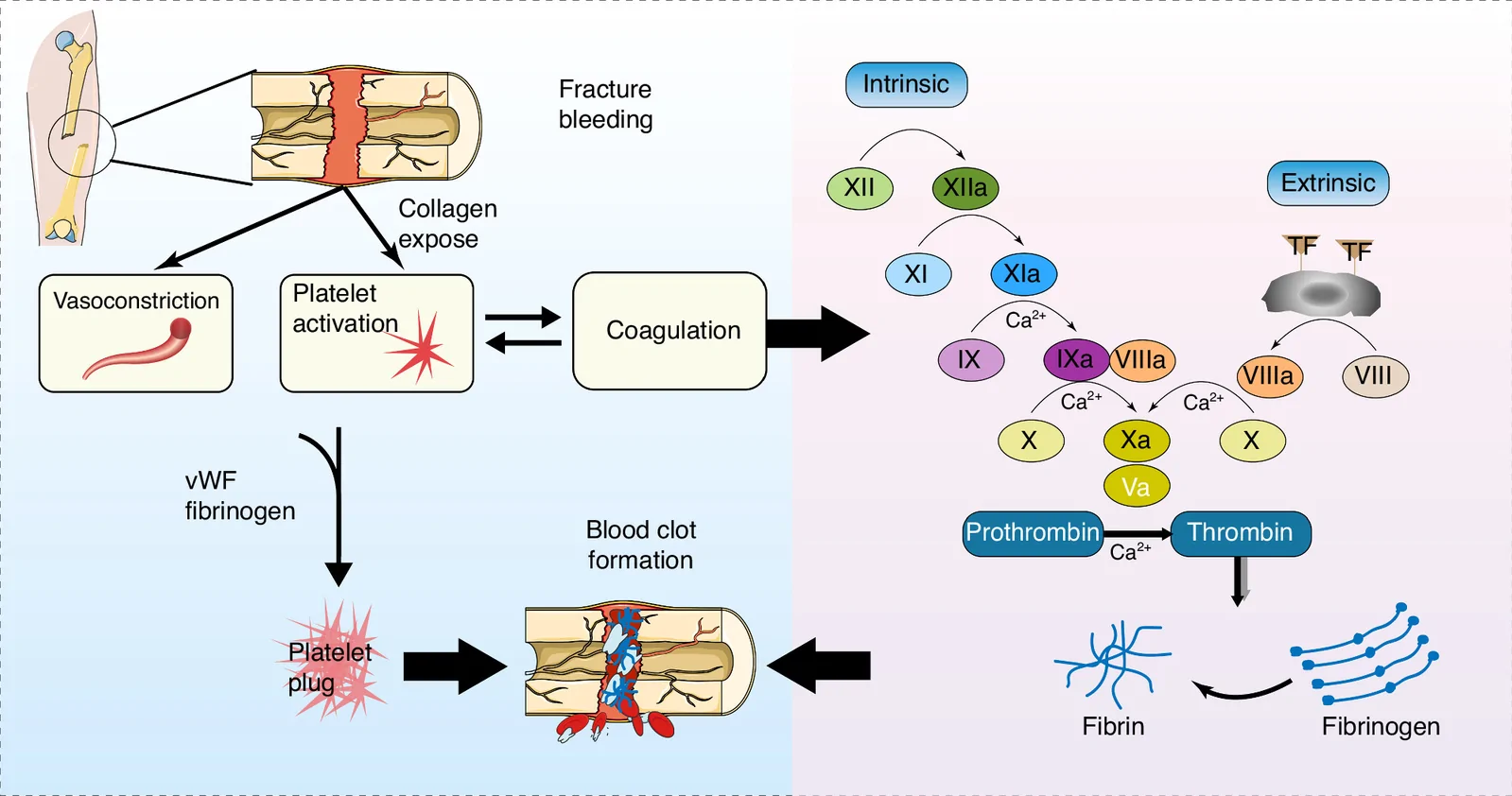
Schematic of the complex mechanism of hemostasis in bone trauma hemorrhage. The bone trauma leads to vascular damage, triggering spontaneous vasoconstriction and tissue factor (TF) in response to bleeding. The exposure of collagen at the injury site promotes platelet adhesion. VWF facilitates platelet adhesion and activation, leading to the formation of platelet thrombi. TF can activate factor VII (FVII) into FVIIa, forming an extrinsic tenase complex (TF- FVIIa). This complex converges to the extrinsic coagulation common pathway initiated by coagulation factor Ⅻ, where factor X is converted to Xa with the help of Ca^2+^ and phospholipids (PL). FXa further activates factor V to generate the prothrombinase complex (FXa-FVa), which catalyzes the conversion of prothrombin to thrombin. Finally, thrombin catalyzes the conversion of fibrin from fibrinogen, leading to the formation of a fibrin clot and achieving hemostasis

The rapid formation of blood clots in the early minutes following bone trauma mainly involves three events (Fig. 1): (1) vascular contraction, (2) platelet aggregation leading to thrombosis, and (3) fibrin clot formation.^20^

#### Vascular contraction

Physiological hemostasis is initiated when small blood vessels at the injury site and in the surrounding areas undergo vasoconstriction, thereby reducing local blood flow.^21^ The reduction in blood flow facilitates the formation of platelet thrombi, which further promotes hemostasis. Simultaneously, collagen is exposed to damaged areas, promoting platelet adhesion. Platelet aggregation triggers the release of 5-hydroxytryptamine (5-HT) and thromboxane A2 (TXA2), both of which further promote vasoconstriction and slow blood flow in favor of thrombosis. The severity of the injury correlates with the intensity of vasospasm, which can persist for several minutes to hours, during which platelet aggregation and clot formation occur. These processes occur simultaneously and have a synergistic effect, collectively promoting the progression of hemostasis.^22^

#### Platelet aggregation and thrombus formation

The main blood cell types in a blood clot are erythrocytes (95%), platelets (<5%), and leukocytes (<1%).^7^ Despite comprising only 5% of blood cells, platelets play vital roles in hemostasis by releasing angiogenic and osteogenic growth factors. Following vascular injury, the exposure of subcutaneous collagen activates platelets, which release chemical signals. Some platelets adhere to the subcutaneous matrix within 1–2 s. Platelet adhesion, mediated by vWF, helps identify the injury site, enabling accurate thrombus localization.^23^ Adenosine diphosphate (ADP) released by locally damaged red blood cells and thrombin generated during coagulation further activate platelets, triggering the release of endogenous ADP and thromboxane A2 (TXA2). This process stimulates additional platelets in the bloodstream to aggregate irreversibly, amplifying platelet aggregation.^24^ Platelets accumulate on exposed collagen to form a thrombus, achieving primary hemostasis.^25^

Activated platelet surfaces expose negatively charged phospholipids (e.g., phosphatidylserine), which promote the co-localization and activation of various coagulation factors, leading to the formation of the tenase and prothrombin complexes. These complexes amplify thrombin production, which converts fibrinogen to soluble fibrin. Thrombin-activated factor XIIIa (FXIIIa) further cross-links fibrin into a biopolymer network that stabilizes the clot by holding platelets and other blood components.^26^ Furthermore, activated platelets secrete polyphosphate (PolyP), which enhances clot formation by activating factors XI (FXI) and V (FV), further amplifying thrombin production.^27^ Trapped platelets release numerous growth factors, cytokines, chemokines, and other signaling molecules that regulate the inflammatory phase of wound healing.^28^ More importantly, activated platelets and the substances they release can stimulate the proliferation and migration of osteogenic cells.^29^

#### Fibrin clot formation

Platelet thrombus formation is followed by secondary hemostasis, during which the coagulation cascade is activated. This process converts unstable fibrinogen into a stable fibrin network, forming a clot that is fixed at the injury site.^30^ The coagulation cascade can be divided into intrinsic and extrinsic enzymatic pathways, which converge to form clots and reinforce primary hemostasis. The intrinsic pathway is activated when coagulation factor XII (FXII) binds to a negatively charged surface, initiating a downstream proteolytic cascade that activates other coagulation factors, ultimately leading to the activation of coagulation factor X (FX). All factors in this pathway are derived from the blood. The extrinsic pathway is triggered by tissue factor (TF), which activates coagulation factor VII (FVII). This initiates and amplifies the coagulation cascade and further activates FX.

The intrinsic and extrinsic pathways then merge into a common pathway, where factor Xa (FXa) cleaves prothrombin to form thrombin. Thrombin, in turn, catalyzes the conversion of fibrinogen to fibrin monomers in the presence of calcium ions (Ca^2+^) and phospholipids (PL). These fibrin monomers then crosslink into a stable fibrin network, facilitated by the action of activated factor XIII (FXIII) and Ca^2+^. In addition to platelets, various blood cells and proteins are incorporated into the fibrin network, forming a stable blood clot.^31^

The network-like structure of fibrin clot plays a crucial role in hemostasis and tissue repair, both through its structural framework and the factors encapsulated within it.^32–34^ Clots with high mechanical strength, high density, and greater resistance to fibrinolysis - characterized by dense fibrin networks- exhibit low porosity and delayed degradation. These characteristics can negatively affect bone regeneration by impeding cell migration. Conversely, less dense fibrin networks may facilitate hemostasis but may not provide adequate support for cell adhesion if they degrade prematurely.^35^ Therefore, an optimal fibrous network with suitable density and degradation speed is essential for both hemostasis and early tissue repair.

During fibrin formation, the clot structure is controlled by pH, ionic strength, fibrinogen, thrombin, calcium, polyphosphate concentrations, and material interactions.^36^ Thrombin activation is a pivotal reaction in the coagulation cascade.^37^ Extremely low concentrations of thrombin ( < 1 nmol/L; < 0.1 U/mL) are sufficient to cleave the fibrinogen and catalyze fibrin polymerization, resulting in turbid fibrin clots composed of thick, loosely packed fibrin chains. Higher thrombin concentrations lead to fibrin clots composed of thinner, tightly packed fibrin chains.^38^

### Cytokines in blood clot

**Fig. 2:**
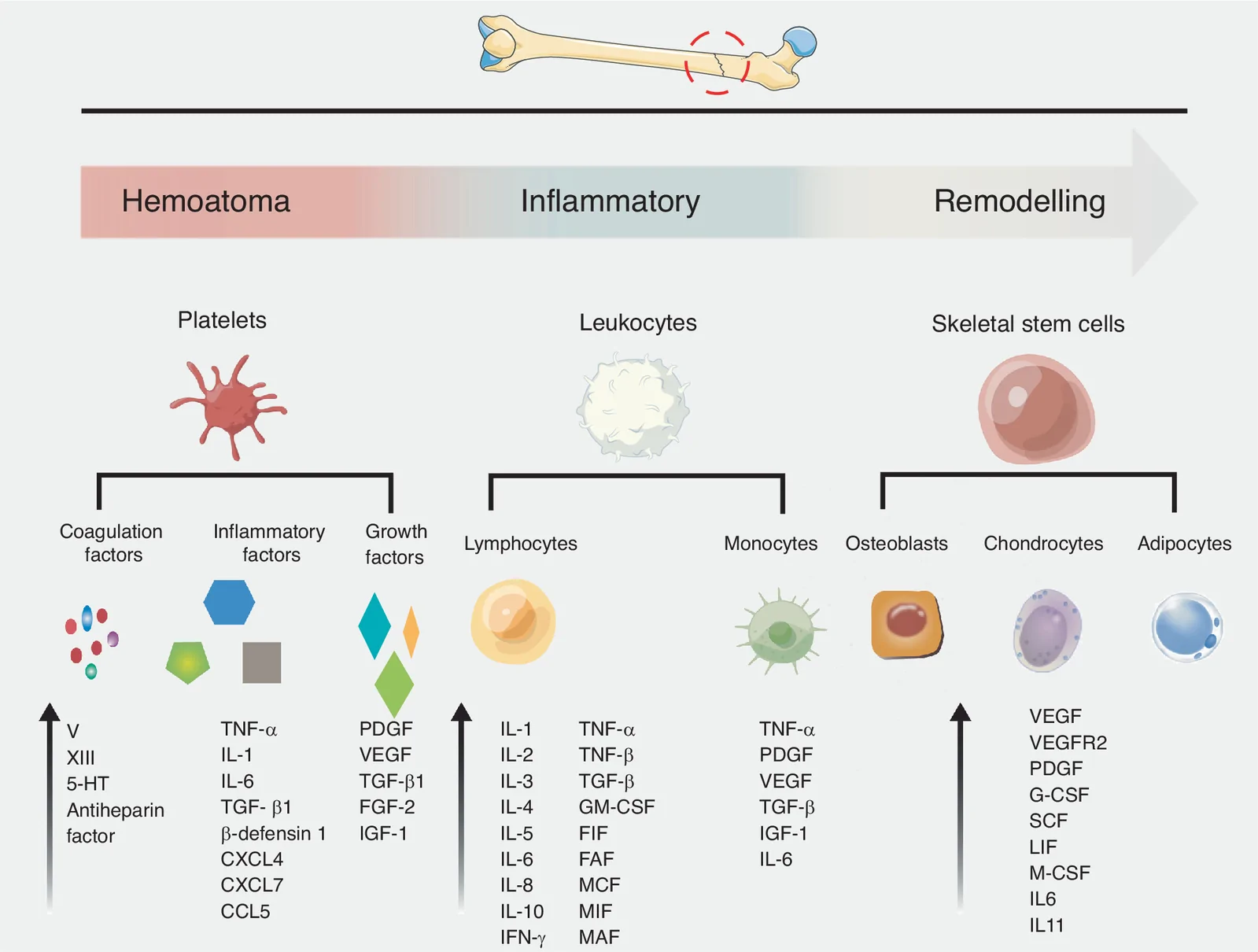
Cytokines released at various stages of bone injury

Blood clots in bone trauma differ from those in soft tissue, as they not only prevent blood loss but also initiate regeneration.^3,7^ These include coagulation components, immune mediators, and osteogenic/angiogenic regulators that collectively orchestrate repair. Three main classes of cytokines in blood clots regulate processes such as hemostasis and tissue repair, as shown in Fig. 2, respectively.

Upon vascular injury, platelets rapidly interact with extracellular matrix (ECM) proteins such as collagen, vWF, and fibronectin, triggering platelet adhesion, aggregation, and activation.^39^ The resultant platelets comprise a range of cytokines, enzymes, and growth factors. These coagulation-related factors include coagulation FV, FXIII, fibrinogen activating factor, lipid-like factor, antiheparin factor, fibrinogen coagulant factor, antifibrinolytic factor, platelet cothromboplastin, anti-thromboplastin, and 5-HT. These factors contribute to blood vessel constriction, thrombin and fibrin formation, and the inhibition of fibrinolysis, activating the coagulation cascade. Once thrombin is generated, it not only promotes platelet activation but also accelerates the production of inflammatory factors by platelets, triggering a cascade effect. Furthermore, platelets directly participate in immune responses by secreting antimicrobial peptides and proteins such as β-defensin 1, thromboxane, CXCL4, CXCL7, and CCL5. These play a role in inflammation and wound repair.

In addition to their hemostatic and immunomodulatory roles, platelets serve as a major reservoir of growth factors that support bone regeneration. These factors include platelet-derived growth factor (PDGF), vascular endothelial growth factor (VEGF), transforming growth factor-β1 (TGF-β1), basic fibroblast growth factor-2 (FGF-2), insulin-like growth factor-I (IGF-I), and platelet factor 4, which stimulate the proliferation and differentiation of undifferentiated mesenchymal cells and osteoblasts.^40,41^ Notably, the release of PDGF-AB and TGF-β1 initiates osteogenesis as early as the stage of platelet aggregation, simultaneously triggering endothelial healing pathways at the injury site^42^ Platelet-rich plasma (PRP) and platelet-rich fibrin (PRF) are reported to promote cell proliferation and bone repair.^43^ Collectively, these platelet-derived factors orchestrate a well-regulated cascade that links hemostasis, inflammation, and tissue regeneration, highlighting the pivotal role of blood clots in initiating and coordinating bone repair.

Leukocyte-related factors represent another important group of cytokines. Leukocytes contribute to the body’s defense mechanisms by secreting a variety of cytokines, which regulate inflammatory and immune responses. Neutrophils, the first inflammatory cells to respond, migrate to the wound site within 24 h and perform phagocytosis to reduce inflammation. Monocyte-derived macrophages respond to changes in the extracellular microenvironment by releasing cytokines and growth factors that mediate cell migration, proliferation, collagen synthesis, and angiogenesis. Among these, interleukin-6 (IL-6) plays a crucial role in all stages of osteogenesis. In addition to regulating the formation of blood clots, IL-6 affects the structure of blood clots by modulating γ-fibrin and participates in angiogenesis during bone repair.^44,45^

Skeletal stem cells (SSCs), capable of differentiating into osteoblasts, chondrocytes, adipocytes, and myocytes, are essential for tissue repair. Their recruitment to blood clots is regulated by inflammatory cytokines (IL-1, IL-6, TNF-α) and enhanced by platelet-derived growth factors (PDGF, TGF-β1), which also promote SSCs proliferation and migration.^46,47^ SSCs secrete factors such as VEGF, PDGF, G-CSF, SCF, LIF, M-CSF, IL-6, and IL-11 to induce osteogenic and chondrogenic differentiation. They also express BMPs to stimulate angiogenesis during bone repair.^48^

Thus, a blood clot formed by blood coagulation gathers cells and factors from the blood and surrounding tissues, creating a specific hemostatic tissue repair microenvironment. Factors released by platelets, leukocytes, and SSCs continuously promote the tissue repair process. The role of these factors in bone repair enhances our understanding of the mechanisms by which blood-derived materials aid in bone repair and offers a viable strategy for their use in early-stage bone repair.

### Initial response at the material-blood interface and the initiation of clot formation

The interaction between hemostatic materials and blood at the material-blood interface is a critical step in the initiation of clot formation. Upon contact with blood, a series of immediate biochemical and biophysical reactions are triggered, ultimately leading to the formation of a stable hemostatic plug. These initial responses not only determine the efficiency and effectiveness of hemostatic materials but also set the stage for subsequent events that regulate clot stability, fibrinolysis, and tissue repair.^49^ A thorough understanding of these early-stage interactions is essential for developing advanced materials with optimal hemostatic properties.

Upon initial contact between a hemostatic material and blood, the adsorption of plasma proteins onto the material surface represents the first critical event.^38^ Key proteins such as fibrinogen, albumin, and various coagulation factors rapidly accumulate on the material surface. The nature and amount of protein adsorption significantly influence subsequent platelet adhesion and activation, as well as the formation of the initial fibrin clot. Fibrinogen, in particular, plays a pivotal role in mediating platelet aggregation and clot stabilization.

Subsequent to protein adsorption, platelets are attracted to the material surface, where they adhere and become activated to express pro-coagulant factors. The interaction between platelet receptors (such as glycoprotein IIb/IIIa) and the adsorbed fibrinogen triggers platelet activation, causing morphological changes, granules release, and the formation of pseudopodia. This activation facilitates the aggregation of additional platelets, leading to the formation of a platelet plug that temporarily seals the bleeding site.^50^ The response of platelets to the material surface is crucial for the efficiency of clot formation and is a key factor determinant of the overall performance of hemostatic materials.

The adsorption of plasma proteins and subsequent platelet activation collectively trigger the coagulation cascade, leading to the conversion of fibrinogen into fibrin. This process begins with the activation of coagulation factors, which ultimately generate thrombin.^51^ Thrombin catalyzes the polymerization of fibrinogen into fibrin, forming a fibrin mesh that stabilizes the platelet plug and provides a scaffold for further clot development. The surface properties of hemostatic material can modulate the activation of the coagulation cascade by presenting specific binding sites for key coagulation factors or by facilitating the assembly of coagulation enzyme complexes.

### Blood–material interactions

**Fig. 3:**
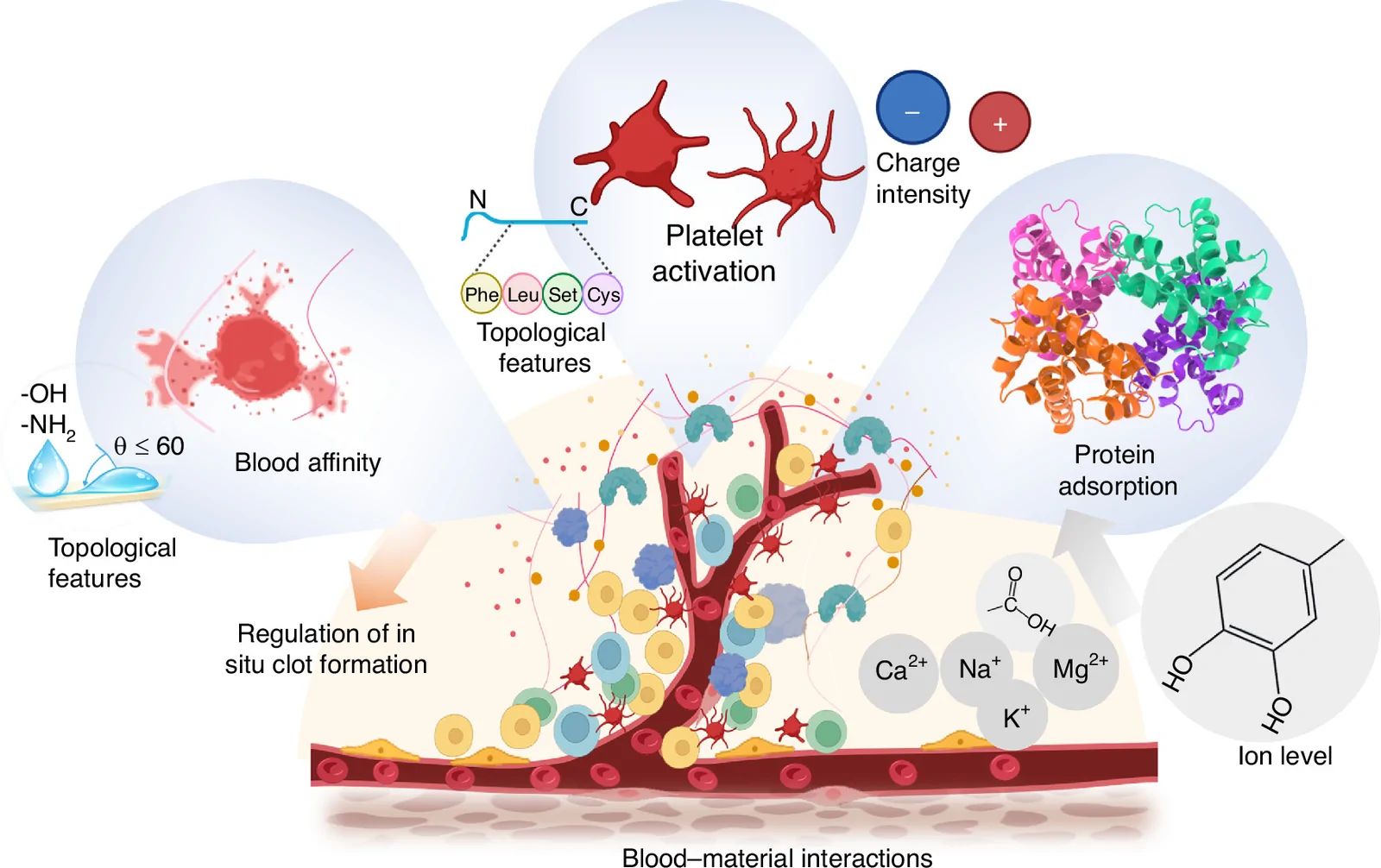
Material–blood interactions promote blood clot generation and in situ hemostasis

The surface characteristics of biomaterials affect their interactions with blood, such as blood affinity, protein adsorption, and platelet activation (Fig. 3).^52,53^

#### Effects of material surface properties on blood

The evaluation of materials exposed to blood at the nano-and micrometer scales affects hemocompatibility through surface roughness and topography,^54,55^ as rough surfaces may lead to stronger and faster blood coagulation, providing a stable provisional matrix for bone repair. Hydrophilicity is another major factor governing blood–material interactions. At present, the mainstream view of hemostatic materials integrated into bone defects is that they rely on water absorption to accelerate coagulation. The interaction between a hydrophilic interface and blood rapidly enriches blood cells and platelets, forming a dense layer that promotes clotting.^56^ However, materials with both low water absorption and rapid coagulation characteristics are rare. In the context of bone-implant integration, the interfacial stability is critical; for instance, if the initial fibrin network is loosely attached to the scaffold fibers, the resultant clot may be easily disrupted during skeletal movement, leading to secondary micro-bleeding. Therefore, biomaterial surfaces that ensure robust clot adhesion under physiological conditions are advantageous for bone repair.

**Fig. 4:**
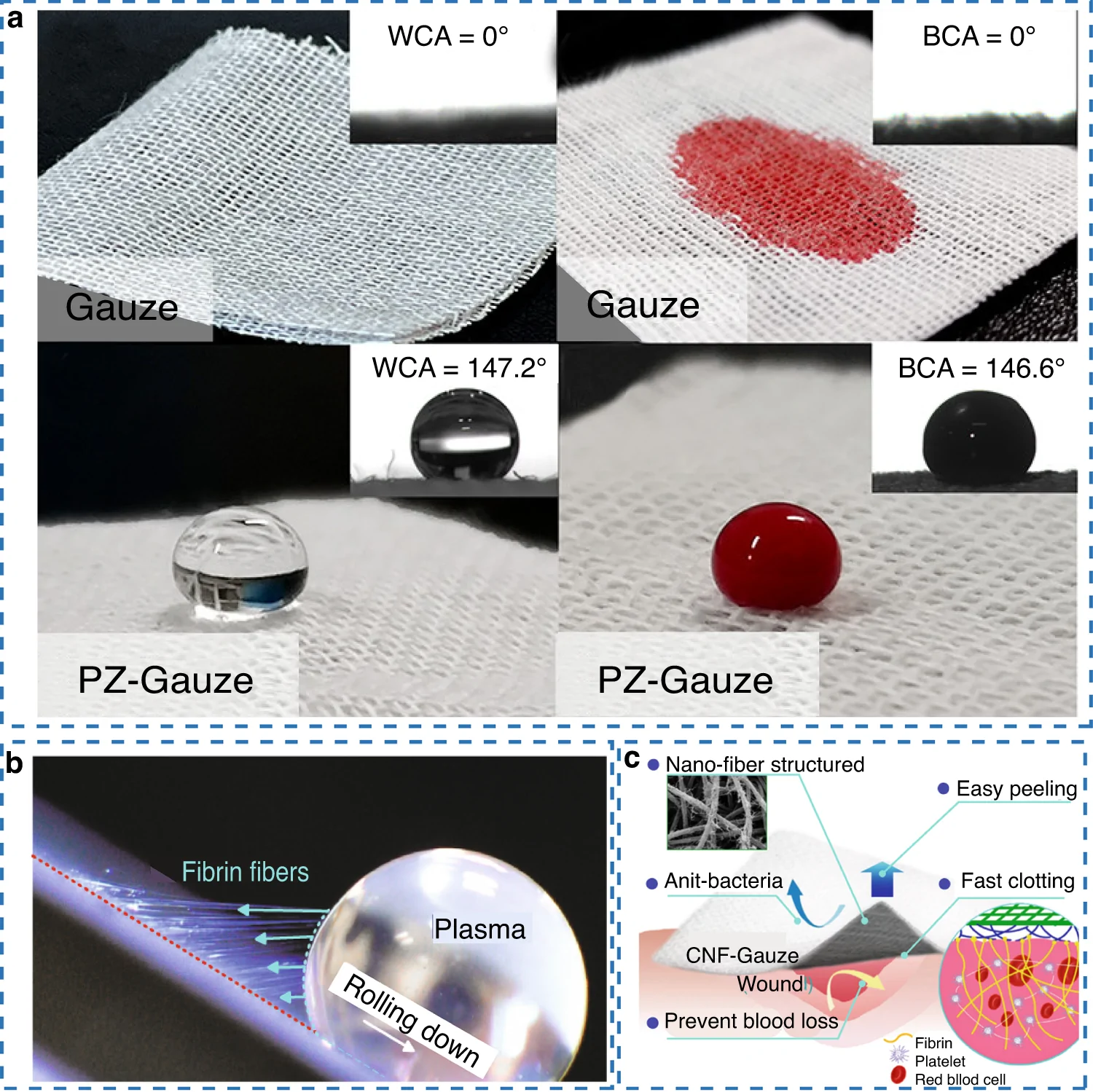
Wettability and blood‑material interaction of functional gauze. **a** Photo of water or blood on the surface of PZ-Gauze and Gauze.^57^ Copyright 2021, American Chemical Society. **b** Fibrin microfilaments generated by platelet poor plasma (PPP) droplet flow at superhydrophobic interfaces.^61^ Copyright 2019, Li, Z et al. **c** Schematic diagram of CNF gauze on the wound surface.^61^ Copyright 2019, Li, Z et al

An example of this interface modulation is seen in modified implant surfaces (Fig. 4a), where surface coatings are used to balance blood affinity and coagulation activity. To address the issue of blood loss, the surface of the gauze was modified with paraffin coating, and a hydrophobic PZ-gauze was prepared. In the case of PZ-gauze, compared to Z-gauze, the coagulation activity was not significantly reduced, while the detachment force was optimized (from 348.8 to 84.7 mN) to avoid excessive mechanical stress on the nascent tissue interface. Furthermore, the PZ-gauze effectively reduced blood loss during treatment.^57^ Improving the hydrophilic properties of materials could significantly enhance their hemostatic effects. For instance, spraying β-chitosan on the surface of porous PCL nanofibers improves fiber hydrophilicity, increasing blood wetting and promoting coagulation.^58^ Recently, researchers discovered superhydrophobic/superhydrophilic materials that can absorb blood effectively, promote clotting, and regulate clotting homeostasis to support the bone repair microenvironment.^59^

In contrast to purely hydrophilic strategies, hydrophobic micro/nanostructures inspired by lotus leaves have also shown hemostatic potential. When blood rolls on ultra-sparse nanofibrous surfaces, fibrin microfilaments can form rapidly and polymerize into a network that traps blood cells and SSCs, even in the presence of anticoagulants.^60^ Using this concept, superhydrophobic carbon nanofibers (CNFs) have been engineered for hemostasis. Micro-air pockets within the blood–CNF contact zone (Fig. 4b, c) reduce direct blood–surface contact, enabling rapid clotting while limiting blood wetting. Notably, CNFs facilitate stable clot formation, reduce blood loss, and significantly decrease bacterial adhesion.^61^ However, current studies remain largely limited to non-degradable CNFs, and further development of biodegradable nanofibers may expand the translational potential of hydrophobic surfaces for bone defect hemostasis.

#### Adsorption of plasma proteins and the mechanism of blood clot formation

An accurate understanding of the hemocompatibility of materials is a cornerstone in the design of regenerative implant materials. Although substantial progress has been made in recent years regarding material biocompatibility, the contact between biomaterials and blood can still trigger a range of overreactions by the immune defense system, indicating that the challenge of hemocompatibility remains unresolved.^62^ The hemocompatibility of a material is one of the most fundamental and important criteria for evaluating implantable biomaterials.

Recent reports suggest that blood compatibility is influenced by a combination of factors, including surface roughness, surface energy, surface tension, wettability, and fiber diameter.^63,64^ The interactions between the material and blood lead to (1) changes in platelets, red blood cells, and white blood cells; (2) the production of activation products in the plasma; and (3) the deposition of proteins and cells on the cell surfaces of the material. Upon exposure to blood, a layer of plasma proteins is rapidly formed on the material surface, and the composition and conformation of this layer are critical for the subsequent activation of the blood cascade system. This protein layer plays a key role in activating leukocytes, monocytes, and platelet functions, while also coordinating the initiation of thrombotic and inflammatory responses on the material surface.^65,66^

Biomaterials influence the composition, quantity, and conformation of the primary protein layer based on the hydrophobic/hydrophilic properties of the surface, as well as the distribution of charges and charged groups. Proteins tend to undergo significant changes when bound to hydrophobic surfaces,^67^ resulting in a higher density of protein deposition on hydrophobic surfaces.^68^ Numerous studies have shown that coating the material surface with polyethylene glycol (PEG) reduces the level of non-specific protein adsorption, which alters the material’s ability to activate the coagulation system and influences subsequent platelet and cell adhesion.^69^ In addition, surface charge also modulates protein–ion interactions; for example, negatively charged PLGA microspheres can adsorb Na⁺, K⁺, and Mg²⁺, with adsorption levels correlated with the zeta potential strength of the material.^70^

Among the adsorbed proteins, fibrin (ogen) and fibronectin are particularly relevant to clot architecture and stabilization. Fibronectin contains many molecular binding domains in the plasma, such as the N-terminal domain, which can play a role in coagulation.^71^ This domain contributes to the cross-linking activity within fibrin clots by forming ε-(γ-glutamyl)-lysyl bonds, catalyzed by thrombin-activated coagulation factor XIII (FXIIIa, plasma transglutaminase).^72^ This cross-linking increases both the thrombus size and platelet adhesion.

Beyond nonspecific adsorption, maintaining the stable attachment of functional proteins and growth factors on biomaterial surfaces remains a key engineering challenge. For example, untreated polycaprolactone (PCL) fibers show poor affinity for protein immobilization. However, surface modification with carboxyl (–COOH) groups significantly enhances protein binding, thereby improving SSCs adhesion. Covalently bound COOH-modified PCL fibers have been shown to stabilize platelet-derived growth factors, promoting increased SSCs adhesion and proliferation.^43^

**Fig. 5:**
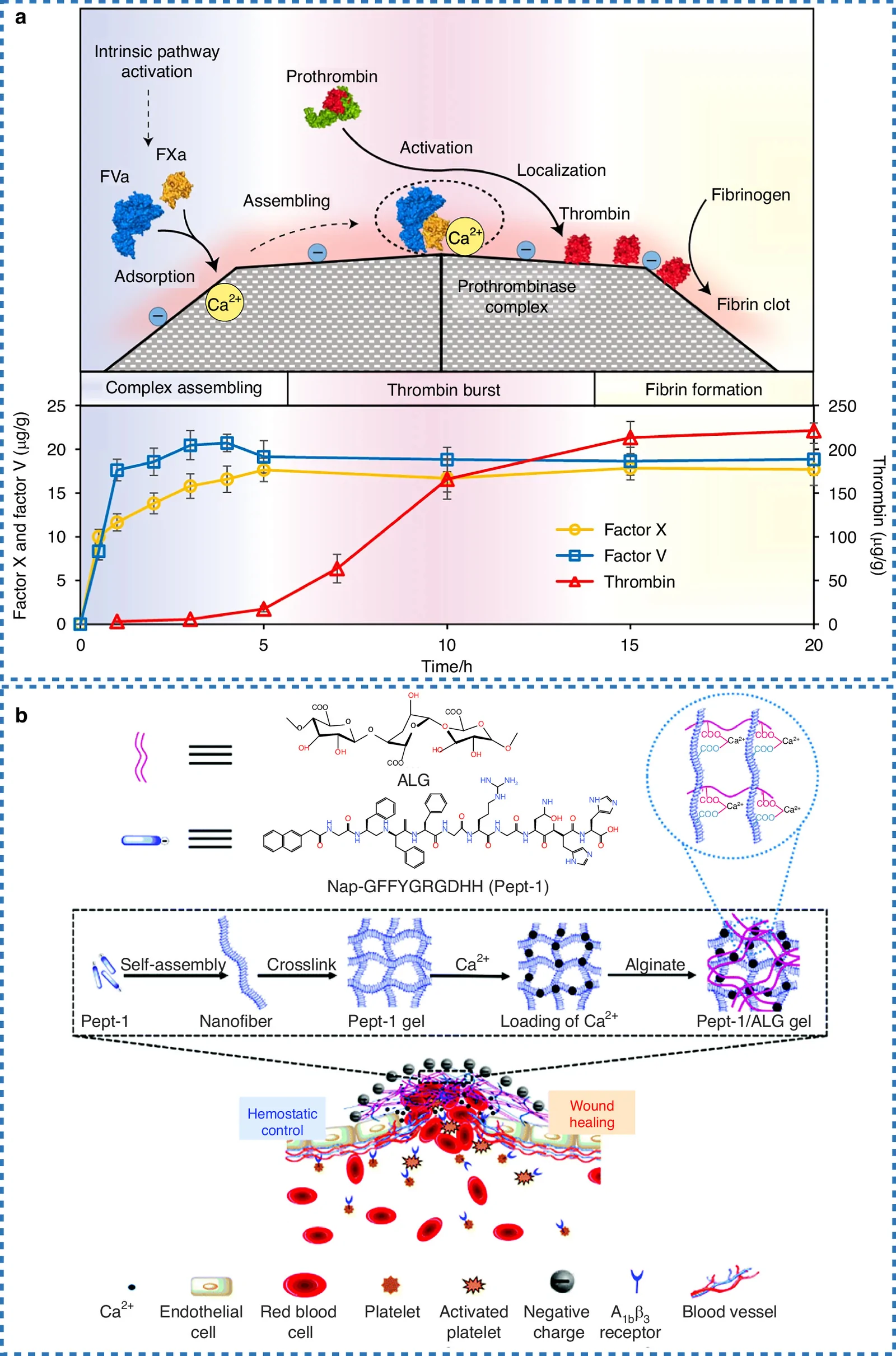
Illustrations of material‑mediated hemostatic mechanisms. **a** Schematic diagram of the assembly of coagulation factors X and V on the zeolite surface.^74^ Copyright 2021, Shang, X et al. **b** Schematic diagram of a co-assembled hydrogel of cell adhesion peptide coupling (Pept-1) and alginate (ALG) for hemostasis and wound healing.^80^ Copyright 2019, Royal Society of Chemistry

Material properties critically govern the adsorption of fibronectin, altering the availability of its N-terminal domains and thus regulating the initiation of coagulation. In addition, surface modifications can facilitate the exposure of specific peptide sequences that directly engage the coagulation process.^73^ Therefore, the type, concentration, and conformational arrangement of the adsorbed proteins are decisive determinants of thrombus formation, platelet recruitment, and immune cell activation. Despite this, the interaction between material surfaces and functional proteins remains largely unresolved. The generally accepted mechanism of hemostasis is the concentration of protein coagulation factors through water adsorption, which simplifies the hemostasis process. Recently, Shang et al. demonstrated that zeolite effectively initiates and propagates the coagulation cascade via an intrinsic pathway (Fig. 5a), intrinsically trigger the coagulation cascade: they template the assembly of the prothrombinase complex (factor Xa/Va), nucleate platelet-like coagulation initiation, and elicit a burst of thrombin generation that drives rapid fibrin polymerization.^74^ These findings establish that rationally designed artificial catalysts can restore or even amplify the body’s natural clot-forming machinery.^67^

#### Adhesion, activation and pro-repair of platelets

Platelets are the first responders to vascular injury and play a pivotal role in a variety of physiological processes, including signal transduction, hemostasis, inflammation, wound healing, and tissue regeneration.^41,42,75^ Therefore, it is essential that platelets accurately accumulate at the site of bleeding to promptly initiate hemostatic and regenerative responses. Platelet adhesion is primarily mediated by integrins on the platelet surface, particularly the major integrin αIIbβ3, which is indispensable for normal platelet function. This integrin binds to a range of ligands containing the arginine-glycine-aspartate (RGD) sequence.^76^ These interactions initiate coagulation via both adhesive mechanisms and the activation of locally produced thrombin and adenosine diphosphate (ADP). Pharmacological agents targeting αIIbβ3 integrin inhibit its interaction with fibrinogen and other RGD-containing ligands, thereby modulating platelet aggregation and thrombosis. Such drugs have demonstrated effective antithrombotic activity with a relatively low risk of inducing bleeding.^76^

Compared to cutaneous wounds, bleeding caused by bone trauma is more complex due to the involvement of hard tissue discontinuities, which often result in more severe hemorrhage and may necessitate the use of hemostatic repair materials. Consequently, material-based strategies that promote precise platelet aggregation and activation are particularly crucial in the management of bone-associated bleeding.^77^

Nature provides a diverse array of biomaterials with significant applications in hemostasis and bleeding control. Many of these naturally derived materials promote blood coagulation by enhancing platelet aggregation and activation. Among them, collagen and gelatin based hemostatic agents, such as Avitene, Helistat, Instat, GelFoam, and FloSeal-facilitate platelet activation through specific receptor interactions. These materials bind to vWF via the GPIbα receptor on platelet surfaces and directly to collagen through GPIa/IIa and GPVI receptors.^78,79^ As such, collagen plays a direct role in platelet recruitment and activation, inspiring the widespread use of bio-derived collagen in hemostatic material development.

Alginate-based materials like Algosteril, which contain negatively charged uronic acid chains, undergo gelation upon exposure to divalent cations such as Ca²⁺. This chelation process is believed to underlie their hemostatic effect.^74^ Calcium ions also function as essential cofactors in platelet activation and multiple steps of the coagulation cascade (Fig. 5b).^80,81^ Similarly, chitosan-based materials promote platelet adhesion, activation, and aggregation through the mobilization of Ca²⁺. In contrast, zeolite-based agents, such as QuikClot powder and modified QuikClot gauze, induce platelet activation by releasing calcium ions, thereby triggering factor XII (FXII) and initiating the intrinsic coagulation pathway.^82^

The significance of early blood-material contact is well recognized—not only for activating platelets to initiate healing but also for serving as a provisional matrix that temporarily supports tissue repair before new bone forms.^83^ Our research has shown that platelet activation levels are significantly influenced by the surface morphology of the material, as interaction with the material surface alters the release of platelet-derived factors. Lena et al. investigated the mechanisms underlying platelet activation using calcium phosphate (CaP) as a reference and found that surface topography, including micro- and nanotexture, plays a more critical role in platelet activation than the chemical presence of Ca²⁺ and PO₄³⁻. Surfaces with similar microstructures induced comparable levels of platelet activation, regardless of chemical composition.^84^ Moreover, the surface charge of the material is closely associated with the degree of platelet activation: the more positively charged the surface, the stronger the activation response. Positively charged surfaces also enhance the adsorption of fibrinogen, further promoting platelet adhesion and aggregation.

Importantly, these surface parameters rarely act in isolation in practical biomaterials. Surface roughness and micro/nanotopography can amplify the functional consequences of wettability by increasing the effective contact area and stabilizing interfacial water layers, thereby accelerating protein adsorption and fibrin deposition. Meanwhile, surface charge modulates ion-protein interactions and can further bias the conformational presentation of adsorbed fibrinogen and fibronectin, ultimately tuning platelet adhesion and activation. Therefore, clot behavior at the material interface should be understood as the integrated outcome of coupled roughness-wettability-charge effects rather than a single-variable response.

### Mechanisms of fibrin clotting homeostasis regulated by materials

The formation of a fibrin clot marks the culmination of the hemostatic process, and maintaining its mechanical integrity is critical for sustaining hemostasis and preventing rebleeding. Numerous studies have demonstrated that platelets can enhance the stiffness of the clot by facilitating fibrin fiber cross-linking and contraction.^85^ However, excessive thrombotic rigidity should not be pursued indiscriminately, as this may interfere with normal tissue regeneration and vascular remodeling.

Once bleeding is controlled, the body initiates fibrinolysis through the coordinated action of tissue plasminogen activator (tPA) and urokinase-type plasminogen activator (uPA), which are primarily secreted by endothelial cells and macrophages. These enzymes convert plasminogen into plasmin, which hydrolyzes fibrin into degradation products that possess immunomodulatory and chemotactic properties, contributing to wound healing and resolution of inflammation.^79^ The fibrinolytic system is tightly regulated by a balance of activators (tPA and uPA), inhibitors (plasminogen activator inhibitors, PAI-1 and PAI-2), and antifibrinolytic proteins such as α2-antiplasmin (α2-AP). Disruption of this balance may result in either excessive fibrin persistence (risk of thrombosis and fibrosis) or premature clot dissolution (risk of hemostatic failure and rebleeding). Thus, a fine-tuned regulation of fibrin formation and degradation is essential for spatiotemporal coordination between hemostasis and tissue repair.

Biomaterial-based interventions not only trigger procoagulant responses through activation of the coagulation cascade but also play an active role in modulating fibrinolysis. Nanoparticles have been shown to exert both procoagulant and anticoagulant effects depending on their size, surface chemistry, and stabilizing agents. Silver nanoparticles (AgNPs) with diameters ranging from 10 to 100 nm can upregulate platelet GPIIb/IIIa expression and P-selectin activation.^86^ However, stabilization with citrate was found to reduce integrin-mediated platelet responses to immobilized fibrinogen, limiting clot retraction and mechanical consolidation.^87^ These findings indicate that material properties significantly impacted the initiation and resolution phases of coagulation.

To facilitate rapid identification of the shared molecular nodes at the material–clot interface, key interactions involving fibrinogen/fibrin, platelet integrin αIIbβ3, and coagulation factor XIIIa (FXIIIa) are consolidated into an integrated mechanistic framework. Collectively, these molecules constitute a unified regulatory axis underlying diverse biomaterial systems. Surface-mediated fibrinogen adsorption and conformational presentation determine the availability of cell-binding motifs, which are subsequently recognized by αIIbβ3 to trigger platelet adhesion and activation. In parallel, FXIIIa-mediated fibrin crosslinking, potentially modulated by local ionic microenvironments and Ca^2+^ availability, reinforces fibrin network stability and mechanical integrity. Through this coordinated molecular cascade, material surface properties are translated into clot architecture and downstream osteoimmune signaling outcomes.

Taken together, biomaterials regulate fibrin clot homeostasis through coordinated control over fibrin assembly, platelet-fibrin interactions, FXIIIa-mediated crosslinking, and the kinetics of fibrinolysis. This balance is essential for achieving rapid hemostasis while preserving a permissive clot niche for subsequent remodeling and bone regeneration.

## Clot-modulating biomaterial strategies for osteogenesis

Building on the aforementioned physiological mechanisms of clot formation and the principles of biomaterial-mediated clot regulation, this chapter first clarifies the central role and osteogenic potential of the blood clot throughout the bone repair process. Subsequently, it systematically delineates how biomaterials can target and modulate clot behavior to achieve a synergistic coupling between hemostasis and osteogenesis, ultimately leading to material design strategies that integrate clinical translational value with enhanced osteogenic efficacy.

### Effect of blood clot on bone repair

Bone possesses the inherent ability for self-repair after injury.^88^ Local injury rapidly forms platelet-fibrin clots to seal the wound. Notably, the blood clot serves dual functions: it acts as physical support for SSCs proliferation and differentiation, and as a temporary reservoir for multiple growth factors that facilitate bone regeneration^44^. The blood clot connects the ECM to bone tissue, thereby triggering a subsequent inflammatory response.^89^ As elaborated in section “Cytokines in blood clot”, the growth factors in blood clots exert pivotal roles in promoting the proliferation and differentiation of osteogenic chondrocytes and angiogenesis, which are the core processes of bone repair. Additionally, the blood clots provide a bone immune microenvironment, with activated platelets releasing pro-inflammatory factors such as TNF-α, IL-1, and IL-6. These factors promote the recruitment and activation of neutrophils and monocytes, leading to macrophage maturation and inflammatory polarization.

In recent years, non-invasive treatments including acoustic waves, electromagnetic fields, mechanical stimulation, and photobiomodulation (PBM) have been explored to optimize blood clot function and accelerate bone defect repair. Studies have demonstrated that low-power laser biostimulation significantly enhances the regenerative potential of blood clots, nearly doubling newly formed bone volume density.^90^ Similarly, physiological mechanical stimulation (20% strain at 1 Hz) has been shown to enrich VEGF within the clot matrix.^91,92^ This enrichment activates the VEGFR2 signaling pathway in endothelial cells, promoting angiogenesis and subsequently driving the differentiation of skeletal progenitors.

In conclusion, the trauma-induced clot is not merely a hemostatic plug but an active bioreactor that regulates the initial phases of bone regeneration. External stimulation of the clots can alter the effectiveness and rate of bone healing; however, the mechanisms by which these stimuli influence bone healing require further investigation. Additionally, the impact of external stimuli on bone repair is limited, and critical-sized bone defects are challenging to repair using spontaneous healing alone. Therefore, the clots modulation for bone regeneration during bone trauma recovery warrants further investigation.

### Biomaterial modulation of blood clot formation

Collectively, these findings confirm that the blood clot functions as an instructive bioactive niche rather than a passive byproduct of coagulation. By orchestrating the architecture, biochemical composition, and functional properties of the clot, biomaterials can further enhance its osteogenic potential, effectively surmounting the innate physiological constraints of natural clot-mediated repair.

Critical-size bone defects are common in clinical practice, difficult to treat, and significantly impact patients’ health and quality of life. Current treatments, such as autologous and allogeneic bone grafting, result in unavoidable tissue damage and rejection problems. However, the development of tissue engineering has provided a reliable means to address such problems. With the development of research, three iterations of bone-replacement biomaterials have been developed. The first generation of biomaterials includes metals (e.g., titanium or titanium alloys, stainless steel, cobalt-chromium alloys), synthetic polymers (e.g., polymethyl methacrylate, Teflon), and ceramics (e.g., aluminum oxide, zirconium oxide). These materials have significant limitations due to their non-degradability. Second-generation biomaterials include synthetic and naturally derived biodegradable polymers (e.g., collagen and polyester), calcium phosphate (synthetic or derived from natural materials such as coral, algae, and bovine bone), calcium carbonate (natural or synthetic), calcium sulfate, and bioactive glasses. In contrast, third-generation biomaterials aim to combine materials with cellular responses by combining growth factors or using external stimuli to develop biomaterial strategies suitable for specific cellular scenarios.^93^

Research on the osteoconductivity, osteoinductivity, osteointegration, and cell-biomaterial interactions of biomaterials is expanding due to the efforts of numerous researchers. However, while much attention has been paid to the regulatory effects of materials on osteocytes and bone tissue, few studies have focused on how tissue engineering materials influence blood clot quality in critical-size bone defects. In fact, the morphology, roughness, hydrophilicity, and pH of different materials can significantly alter the number and function of cells, factors, and proteins in the blood. Using biomaterials to enhance blood clot function and improve the biological effects of bone implants represents a promising avenue for future research.

We examined the key determinants of blood clot formation in bone-trauma wounds, with particular focus on the fibrin network’s dual role as structural scaffold and growth-factor reservoir. By sequestering cytokines and growth factors within its meshwork, the fibrin clot prevents their premature diffusion and ensures sustained, localized signaling. Both the rate of fibrin polymerization and the resulting fiber architecture are modulated by growth-factor loading and by mechanical cues, which together shape fiber diameter, porosity, and network density—and thus govern endothelial-cell and fibroblast adhesion and migration. Indeed, thicker fibrils create larger interstitial spaces and greater mechanical stability, enhancing integrin‑mediated cell ingress and accelerating bone‑healing processes.^94^

To date, many materials, including metallic and inorganic materials, have been used in conventional stent/implant design and manufacturing. Metals such as titanium (Ti)-based alloys, magnesium and its alloys, and other biodegradable metals have excellent properties, being non-toxic, lightweight, strong, biocompatible, and corrosion-resistant, making them ideal candidates for bone implants with promising applications.^95,96^ Studies have shown that the complex surface structure of Ti scaffolds promotes more extensive fibrin clot formation and greater aggregation of red blood cells.^97^ Hydroxyapatite (HAp) is the primary inorganic component in human and animal bones. Compared to other ceramic biomaterials, hydroxyapatite has a specific adsorption capacity for plasma proteins such as fibrinogen,^98–101^ complement factor C3 (C3), and apolipoprotein D (Apo D), which influences its in vivo behavior, including complement activation, platelet activation, coagulation, and cell adherence.^102^ Similar to hydroxyapatite, tricalcium phosphate (TCP) is widely used as an artificial biomaterial due to its high biocompatibility, bone conductivity, and superior degradation properties.^103^ The released calcium ions promote bone tissue mineralization and enhance bone regeneration. Among the biomaterials that regulate coagulation and blood clot formation, these two classes are the most extensively studied.^29,32,104,105^

### Surface modifications modulated the blood clot for osteogenesis

Building upon the mechanistic insights into how hemostatic materials dialog with blood components described in section “Blood clot formation and mechanisms”, the modulation of blood clots induced by biomaterials offers a powerful strategy to enhance bone regeneration. Hemostatic responses particularly those involving fibrin formation, platelet activation, and the adsorption of growth factors can be fine-tuned by material properties to favor not only rapid hemostasis but also downstream tissue repair. Specifically, material architecture encompassing pore size, scaffold design, and topographical features, serves as a critical structural parameter that directly dictates clot-mediated osteogenic outcomes. These blood–biomaterial interactions at the material-blood interface provide a novel design platform for multifunctional biomaterials that couple bleeding control with osteogenesis.

Although this review discusses biomaterials by category, their practical selection is ultimately governed by a limited set of shared design priorities, including degradability, mechanical contribution, clot regulation efficiency, and defect-specific requirements. To facilitate rapid comparison and strengthen the translational value of this review, we summarize representative biomaterial classes and their dominant clot-modulating mechanisms, together with their typical clinical scenarios and limitations (Table 1). Notably, hybrid systems integrating engineered scaffolds with blood-derived matrices increasingly represent a rational approach to bridge immediate bleeding control with sustained osteogenic support, particularly in complex trauma and critical-sized defects.

**Table 1:** Comparison of representative biomaterial classes for clot regulation-guided bone regeneration

Biomaterial class	Typical examples	Dominant clot-regulation lever	Degradability	Mechanical contribution	Best-fit clinical scenarios	Key limitations	References
Metals	Ti, Ti alloys, stainless steel, CoCr, Mg-based alloys	Protein adsorption + platelet adhesion via surface physicochemical cues	Ti: non-degradable; Mg: degradable	Excellent (Ti), moderate (Mg)	Load-bearing fixation; small defects; implant-associated regeneration	Ti: poor degradability; limited clot integration; Mg: rapid corrosion if uncontrolled	^114^
Calcium phosphate ceramics	HA, β-TCP, BCP	Ca²⁺/PO₄³⁻ ion-mediated fibrin assembly + adsorption of fibrinogen	Moderate to high (composition-dependent)	Moderate (brittle)	Medium/large defects; osteoconductive filling; defect sites requiring bone ingrowth	Brittleness; weak handling; performance depends on porosity and dissolution	^32,117,120,121^
Bioactive glass	45S5, borate glass	Rapid ion exchange (Ca²⁺, Si species) + microenvironment alkalization	High (especially borate)	Moderate	Irregular defects; antibacterial-demanding environments; vascularization-limited defects	pH burst; fragile; cytotoxicity at high dissolution rates	^126^
Synthetic polymers	PLA, PLGA, PCL, PEEK, PU, PEG-based	Tunable surface charge/wettability + controllable degradation products	High (tailorable)	Low–moderate	Injectable/printable scaffolds; minimally invasive delivery; controlled architecture	Weak intrinsic bioactivity; may require surface functionalization	^70^
Natural polymers/hydrogels	Gelatin, collagen, alginate, chitosan, hyaluronic acid	Water absorption + clot integration + growth-factor retention	High (enzymatic)	Low (unless reinforced)	Irregular defects; hemostatic sealing; filling large cavities; bleeding; deep wounds	Weak mechanical support; batch variability; fast degradation unless crosslinked	^141,173^
Blood-derived matrices	PRP, PRF, fibrin glue, autologous clot	Native fibrin network + endogenous cytokine reservoir	High (physiological fibrinolysis)	Low	Small-to-medium defects; adjuvant therapy; patients with normal coagulation	Donor variability; limited tunability; inconsistent clinical outcomes	^187,194,195^

**Fig. 6:**
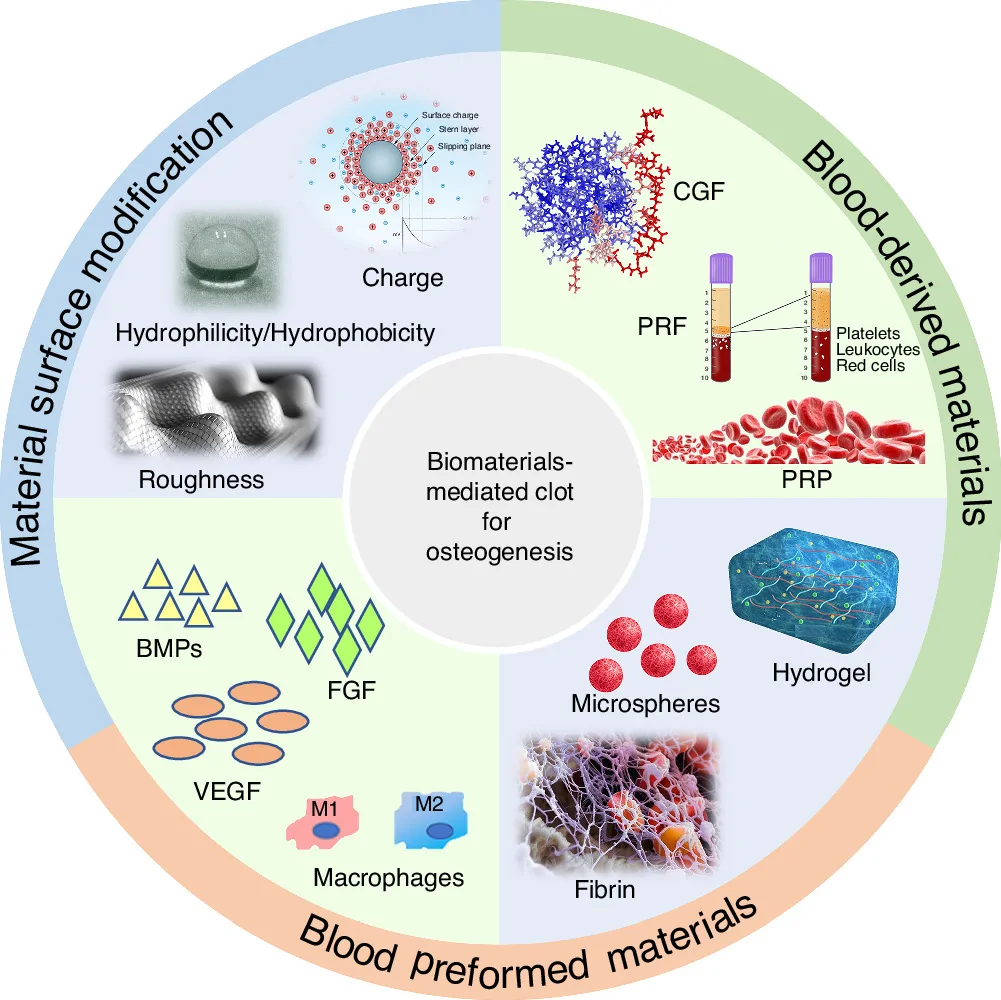
Blood clot-modulating biomaterials for osteogenesis

From an engineering perspective, current approaches to regulate blood clots for bone repair can be broadly distilled into three representative directions: (i) surface physicochemical modulation to steer protein adsorption, platelet activation, and fibrin assembly; (ii) blood-derived matrix construction to create regenerative clot analogs; and (iii) cell-component integration to amplify immune–osteogenic crosstalk within the clot niche. An overview of these strategies and their mechanistic pathways is provided in Fig. 6.

Beyond serving as a provisional matrix, the blood clot functions as an instructive immunoregenerative niche that is highly sensitive to biomaterial cues.^106^ A conceptual regulatory chain can be delineated: surface properties (e.g., charge, wettability, roughness, and ion release) govern the initial adsorption of plasma proteins and platelet–fibrin interactions, thereby shaping fibrin fiber organization and clot permeability. These clot-level features, in turn, regulate early innate immune events, ultimately determining the balance between inflammatory amplification and pro-regenerative signaling to support angiogenesis and osteogenesis. Therefore, rational clot modulation should not aim at clot stabilization alone, but rather at orchestrating a coordinated “material–clot–immune–bone” axis. The following sections will detail how specific surface modifications can be harnessed to achieve this hemostasis-to-osteogenesis synergy.

#### Fibrin structure modulation

Fibrin behavior at the bone-biomaterial interface is closely governed by surface roughness and wettability. Milillo et al. utilized titanium foil to guide bone regeneration (GBR).^107^ The results showed that titanium foil binded to the clot as a protective barrier to avoid resorption. In addition, titanium membranes were tolerated better than non-absorbable alternatives, and cartilage maturation with new bone formation was observed 6 months after maxillary rehabilitation. Surface roughness, in particular, plays a pivotal role in osseointegration by promoting the adsorption of platelets, proteins, and growth factors.^108^ Titanium surfaces with moderate roughness (Ra 2–4 μm) facilitated faster bone-to-implant contact than smoother surfaces.^109–111^ Blood clots formed on rough titanium surfaces (Ra ≈500 nm) exhibit denser and more branched fibrin networks, which enhance SSCs migration ≈2.5-fold and increase viability by ≈50% after five days, despite minimal changes in early cell morphology.^110^

Wettability is another surface feature influencing bone repair. Hydrophilic surfaces in contact with blood promote the differentiation of mesenchymal cells and osteoblasts, enhance early matrix mineralization, and accelerate bone regeneration.^108,112^ In a rabbit model, Guilherme et al. found that hydrophilic surfaces of titanium mesh achieved 30% higher bone-to-implant contact (BIC) ratio than hydrophobic surfaces.^113^ Similarly, Kopf et al. developed a titanium surface that was both microroughened and hydrophilic, which resulted in enhanced protein adsorption and fibrin network formation, and ultimately induced the most robust osteogenic response.^108^

**Fig. 7:**
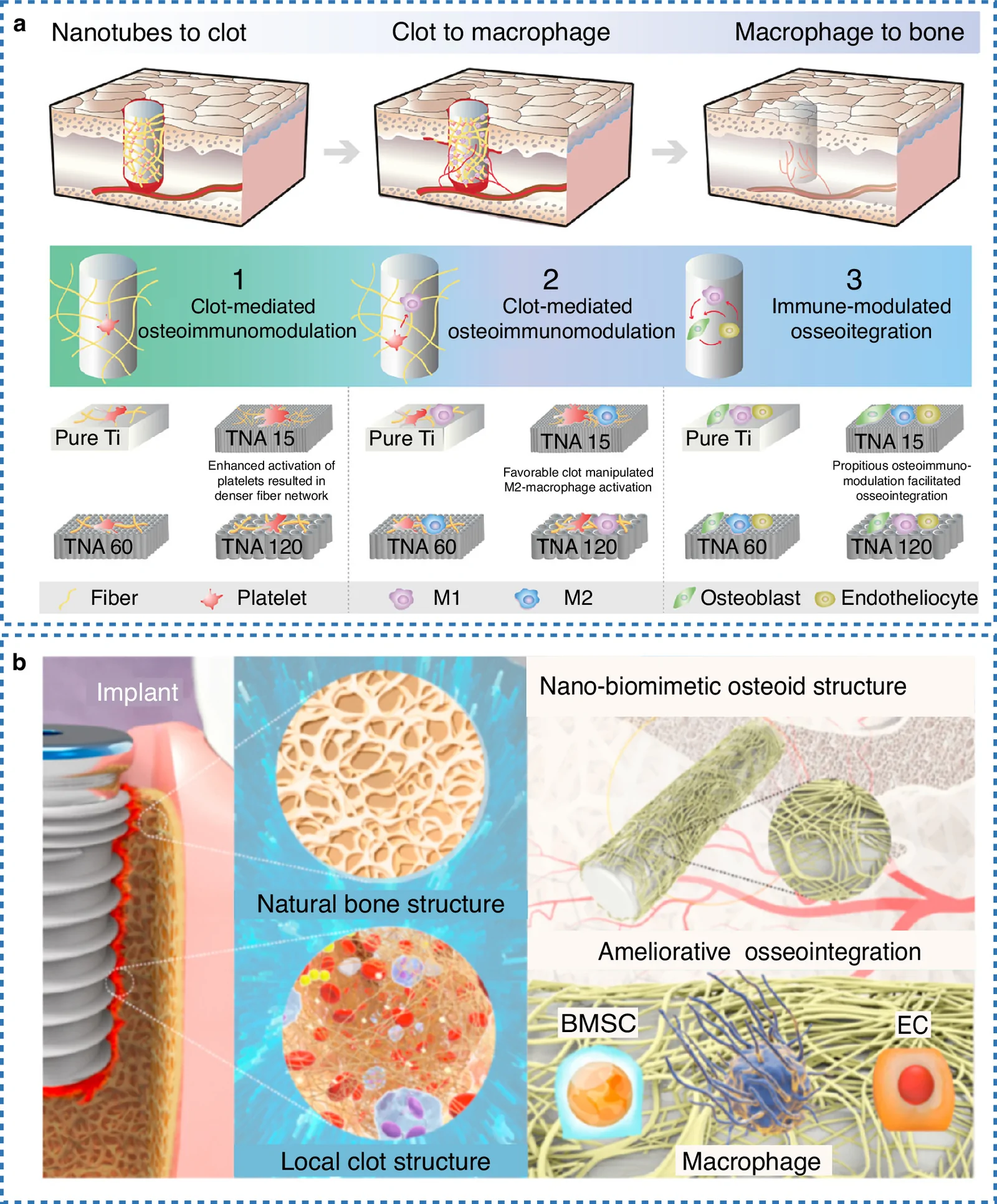
Blood clot‑related responses in implant osseointegration. **a** Illustration of the link among the TNAs, clot, osteoimmunomodulation, and osseointegration.^114^ Copyright 2020, Wiley-VCH. **b** Schematic illustration of the peri-implant blood clot formation, and the de novo bone formation microenvironment during osseointegration.^116^ Copyright 2021, Elsevier

Beyond microtopography and wettability, nanoscale surface features provide an additional layer of regulation over fibrin assembly and immune responses. Titanium nanotube arrays (TNAs) with 15 nm diameter surfaces (TNA15) support denser fibrin networks, approximately double protein adsorption, enhanced platelet spreading, and higher release of PDGF-AB and TGF-β1 relative to flat titanium or larger-diameter TNAs (120 nm) (Fig. 7a).^114^ Clots formed on TNA15 surfaces also modulate macrophage polarization, suppressing inflammatory cytokines (IL-1β, IL-6, IL-8, IL-18, TNF-α), M1 markers (iNOS, CD11C), and osteoclastogenic markers (TRAP, CTSK), while upregulating M2-associated factors (BMP-2, TGF-β1), indicative of a pro-regenerative immune phenotype. Moreover, these clots exhibit distinct long non-coding RNA (lncRNA) profiles associated with osseointegration, highlighting gene-level regulation mediated by nanoscale topography.^115^

Biomimetic micro–nano fibrous structures created via one-step alkaline treatment (5 mol/L NaOH, 25 °C) on titanium surfaces form microporous networks composed of nanofibers that closely mimic the extracellular matrix of the bone-healing environment (Fig. 7b).^116^ These biomimetic surfaces promoted enhanced pseudopodia extension and actin filament adhesion in SSCs, and upregulated key osteogenic genes including RUNX2, ALP, OPN, OPG, BMP-2, VEGF, COL1, and SMAD1/3/4/5/8 within 3 days of culture. In addition, endothelial cell proliferation was increased, suggesting that such micro–nano architectures may simultaneously support osteogenesis and angiogenesis.

**Fig. 8:**
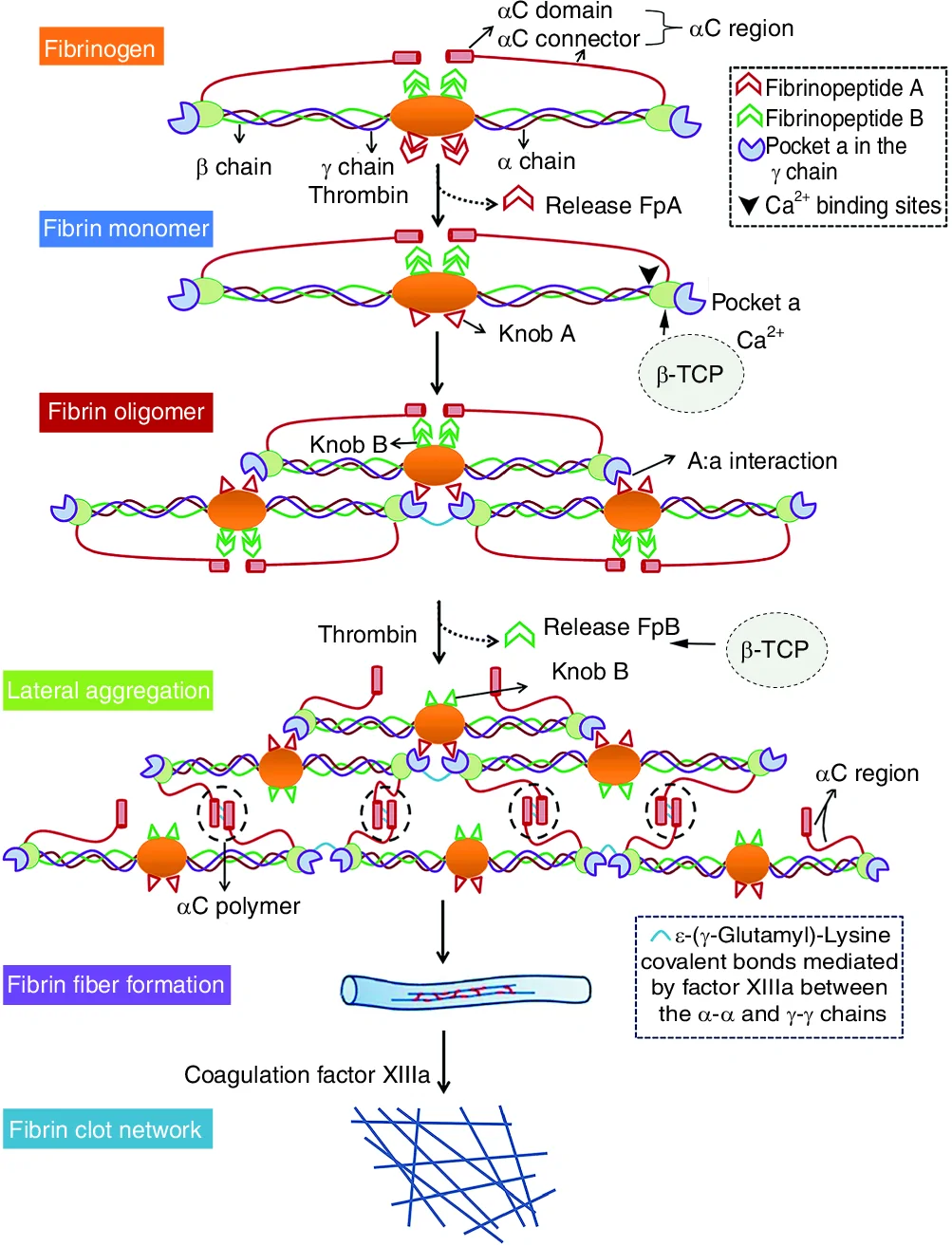
Schematic of polymeric fibrin network formation and β-TCP target sites.^32^ Copyright 2018, Royal Society of Chemistry

Biomaterials can actively influence blood clot formation and interact with blood components during bone graft implantation. β-Tricalcium phosphate (β-TCP) has been shown to modulate fibrin network architecture in a concentration-dependent manner. Wang et al. reported that higher β-TCP concentrations (100–200 mg/mL) produced significantly finer fibrin fibers (≈70–77 nm) compared to low concentrations (≈112 nm at 50 mg/mL) and controls (≈140 nm). At 200 mg/mL, clots also exhibited denser fibrin networks with smaller pore sizes, suggesting that β-TCP provides nucleation sites and local Ca^2+^ release to promote fibrin polymerization along particle surfaces (Fig. 8). These findings indicate that material content can directly modulate clot microstructure and potentially enhance osteogenic properties.^32,117^

Recent studies have investigated how blood-pretreated ceramic bone substitutes influence fibrin network formation and downstream osteogenesis.^32,117^ Hydroxyapatite/β-TCP (HA/TCP) composites with distinct surface topographies were incubated with whole blood, revealing marked alterations in clot architecture. Specifically, HA/TCP induced two fibrin fiber types thread-like (≈63.5 ± 10.1 nm) and net-like (≈76.3 ± 18.3 nm) considerably finer than the dense fibers in native clots (≈126.5 ± 23.4 nm). These structural changes were accompanied by elevated local cytokine-induced neutrophil chemoattractant (CINC) and MMP-8 levels at early time points, and significant upregulation of angiogenic and osteogenic markers (~2.5-fold VEGF and ~1.9-fold ALP increase), highlighting the angiogenic and osteoinductive potential of HA/TCP-mediated clots.^118^

Biphasic calcium phosphate (BCP) materials similarly form cohesive, viscous composites upon mixing with blood, with particle size (80–200 μm) modulating clot porosity, fluid transport, and cell infiltration.^33,119^ Functionalization with fibrinogen (FNG) further improves fibrin polymerization kinetics. Kim et al. demonstrated that FNG-coated BCP retained clotting functionality and enhanced cellular proliferation and adhesion in vivo. Notably, eight weeks post-implantation, FNG40-BCP implants exhibited significantly greater new bone volume compared with unmodified BCP, indicating improved osteoconductivity.^120,121^

Pre-fabricated clot strategies have also been explored. Liu et al. incorporated pre-formed clots within porcine hydroxyapatite (PHA) particles, which modulated fibrin architecture without requiring exogenous stem cells or osteogenic factors. Subcutaneous implantation in rats induced osteogenesis, osteoclast activity, and neovascularization. Specifically, new bone area reached ~32% compared with ~15% in controls, and vessel density increased by ~1.7-fold, closely mimicking outcomes observed in calvaria defect models.^122^

Surface microstructure further regulates clot formation. Wu et al. investigated fibrin networks on calcium magnesium phosphate cement (CMPC) surfaces with needle-like crystalline facets. The microstructure enhanced blood cell adhesion and platelet activation, producing clots with high porosity (≈70.8%) and fine fibrin fibers (≈0.43 ± 0.02 μm). The degradable fibrin matrix supported sustained release of growth factors, promoting cellular migration and adhesion.^13^

Collectively, these studies demonstrate that preformed biomaterial–blood constructs are valuable for evaluating osteoconductivity and highlight the critical role of clot microarchitecture in modulating angiogenesis, osteogenesis, and immune responses. These findings emphasize that tuning fibrin network properties via material composition, particle size, surface structure, or pre-fabrication represents a generalizable strategy to optimize bone regeneration.

Despite the crucial role of blood–biomaterial interactions in bone regeneration, these processes have not been extensively elucidated at the molecular level. In 2021, Jing et al.^123^ applied proteomic analyses to blood clots formed with 80–200 µm BCP particles and found significantly elevated fibrinogen levels, which activate TLR4 signaling in a MyD88- and NF-κB–dependent manner. This activation was mediated by LBP and CD14, linking clot architecture to downstream immune–osteogenic pathways. Beyond molecular signaling, the bulk mechanics of clot–material composites are critical for clinical translation. Calcium phosphate cements (CPCs) incorporated with blood solidify into reticulated fibrin networks with markedly enhanced strength. Mellier et al. reported that two injectable apatitic CPCs achieved compressive strengths of 6.4 ± 0.1 MPa, compared to <2 MPa in earlier blood-calcium phosphate constructs, while maintaining osteoconductivity in vivo.^124,125^

Surface charge modulation is also a promising strategy for influencing early cellular responses. Upon blood contact, biomaterial surfaces undergo protein adsorption and charge redistribution, which subsequently affect further protein interactions and cellular behavior. Numerous studies have underscored the importance of surface charge in forming calcium phosphate layers. Bioactive glass, for instance, exhibited an increase in surface potential from 3.3 to 6.4 mV over 7 days of fibrin adsorption, before stabilizing at ~−14 mV, a charge state that slows Ca–P deposition and modulates protein affinity.^126^ Similarly, titanium and its alloys, while widely used for permanent implants due to high mechanical strength and corrosion resistance,^127,128^ contrast with biodegradable magnesium-based materials that degrade during healing. However, pure magnesium often degrades too rapidly. Alloying with elements of lower electronegativity enhances fibrinogen adsorption energy, whereas more electronegative elements reduce protein affinity, offering a tunable handle for clot formation and regeneration.^129–133^

Moreover, fibrin adsorption can be regulated via nanoporous structures. Bioactive glass with nanopores ≥ 6 nm allows fibrin penetration.^134^ However, differences in pore size also influence solubility and pH, which in turn affect plasma protein interactions. This indirect modulation of fibrin adsorption via nanostructure design holds significant implications for both hemostasis and tissue repair.^135^

**Fig. 9:**
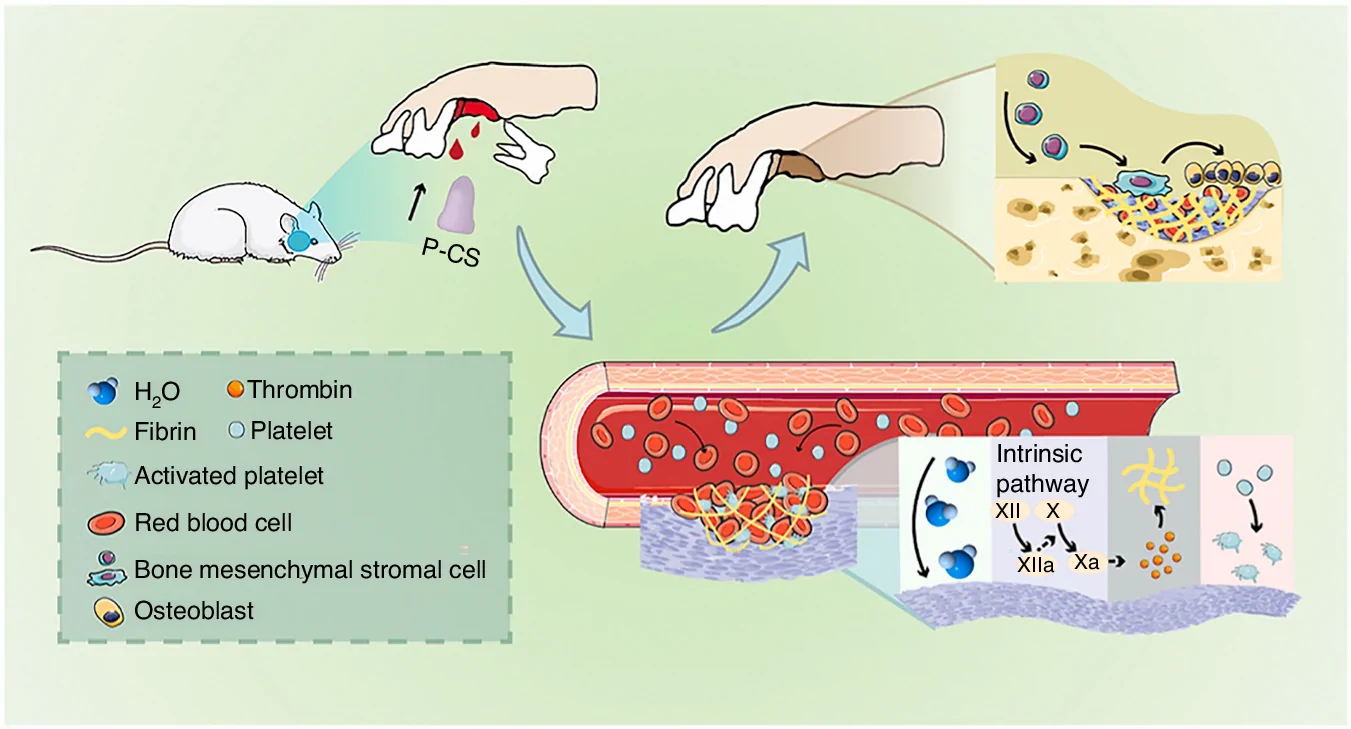
Schematic illustration of the fabrication process and multifunctional hemostasis and osteogenic mechanisms of polyphosphate-collagen scaffold (P-CS).^137^ Copyright 2022, Gu. et al

In addition to physical and chemical cues, material-based biochemical modulation has emerged as a strategy to simultaneously regulate clot integrity and regenerative outcomes. Polyphosphates accelerate coagulation by activating factor XI and boosting thrombin generation, while also promoting SSCs proliferation.^136^ Gu et al. developed polyphosphate-crosslinked collagen scaffolds that generated dense fibrin networks and significantly improved bone repair in tooth extraction models (Fig. 9).^137^ Likewise, sulfated hyaluronic acid (sHA3) cross-linked into collagen scaffolds modulated platelet-rich fibrin behavior, reduced pro-inflammatory cells (CD68⁺, CCR7⁺), and enabled controlled release of VEGF-A and TGF-β1, thereby promoting vascularized bone regeneration.^138,139^

#### Modulating platelet activation

Platelets play a central role in clot retraction, which, if uncontrolled, can lead to tissue detachment and structural defects. To mitigate these issues, biomaterials such as chitin have been incorporated into blood-contacting implants to inhibit clot retraction, reduce secondary bleeding, and improve tissue repair efficiency.^140^ In particular, chitosan glycerol phosphate (CS-GP)/blood implants stabilize clot structure and prevent rapid serum-mediated degradation by interacting with platelet factor 4 and thrombin antithrombin complexes (TAT).^141^

Chitosan also promotes platelet aggregation and exhibits thrombin-like activity, enhancing platelet activation.^142^ Mixed clots of chitosan and platelet-rich plasma (CS-PRP) show elevated cumulative release of key growth factors, including PDGF-AB (~2.5-fold increase) and TGF-β1 (~2.0-fold increase) compared with PRP alone, thereby facilitating hemostasis, articular cartilage repair, bone stem cell recruitment, and subchondral angiogenesis.^143–145^

Surface structural features further modulate platelet behavior. Micro- and nanoscale topographies enhance platelet adhesion (from ~1.2 × 10^4^/mm² on smooth surfaces to ~3.5 × 10^4^/mm² on nanostructured surfaces), activation, coagulation, and pro-regenerative growth factor release, ultimately influencing early inflammatory responses and subsequent tissue integration.^55,112,146^ However, the downstream effects of platelet-material interactions on immune cell behavior, particularly in nanostructured contexts, remain underexplored.

**Fig. 10:**
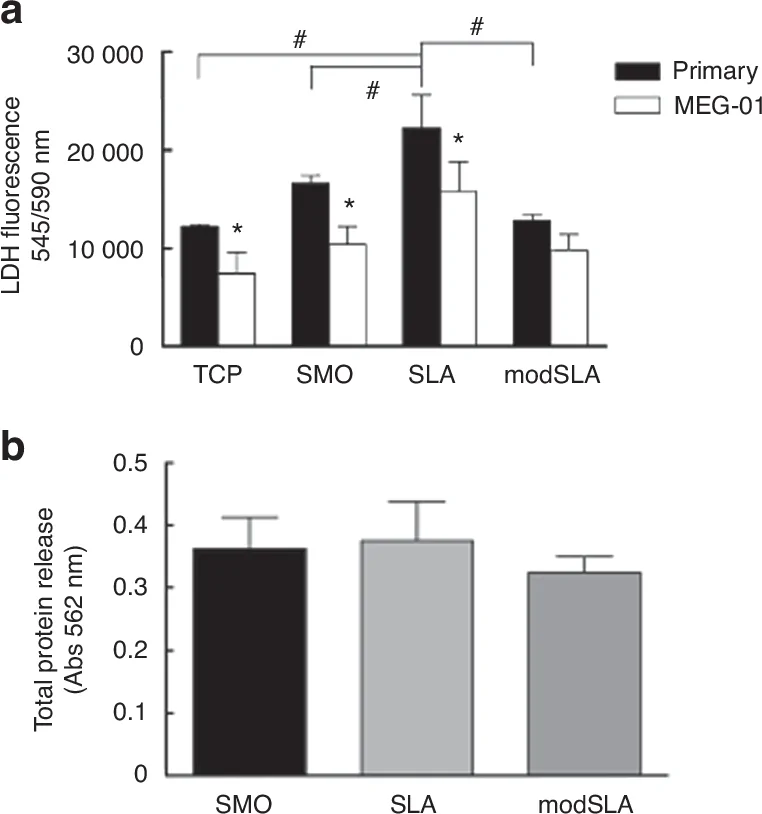
Platelet adhesion and protein release on titanium surfaces. **a** Comparison of the attachment of primary human and megakaryoblast (MEG-01) platelets suspended in media with 10% serum onto control tissue culture plastic (TCP) and the titanium test surfaces: smooth (SMO), microrough (SLA), and hydrophilic microrough (modSLA).^147^ Copyright 2014, Wiley-VCH. **b** Total platelet protein release.^147^ Copyright 2014, Wiley-VCH

In 2013, Dr. Mohammed conducted a study to investigate how platelet activation on titanium surfaces affects cytokine gene expression in macrophages.^147^ Three titanium surface types were examined: smooth polished (SMO), sandblasted and acid-etched (SLA), and hydrophilic-modified SLA (modSLA). While platelet adhesion was reduced on the hydrophilic modSLA surface (1.2–2.3-fold reduction), proteomic analysis showed no significant difference in platelet protein content across the three surfaces (Fig. 10). This suggests that the surface characteristics regulate platelet activation rather than platelet adhesion alone. More importantly, macrophage inflammatory gene expression remained similar among SMO, SLA, and modSLA surfaces in the absence of platelets. However, in the presence of platelets, the modSLA surface significantly downregulated 22 pro-inflammatory and chemokine-related genes (e.g., CCL11, CCL13, CCL16, CCL23, CCL24, CCL25, CCL26…). This indicates that platelet activation on modSLA surfaces may contribute to an accelerated resolution of inflammation.

Together, these findings suggest that biomaterial-induced platelet responses can serve as an upstream modulator of inflammation and tissue integration, ultimately shaping the osseointegration process at bone injury sites.

#### Modulating the selective adsorption and release of growth factors and proteins

Titanium is widely used in bone repair due to its excellent mechanical properties and biocompatibility. Its osteogenic potential can be further enhanced through surface modifications and bioactive coatings. For example, HAp plasma-sprayed onto titanium (HA-Ti) selectively adsorbed (BMP-2), promoting adhesion and proliferation of SSCs on the implant surface.^148^

Beyond coatings, platelet-rich fibrin (PRF)-based strategies can regulate growth factor dynamics. Tunalı et al. developed a titanium-prepared PRF (T-PRF) protocol that forms a mature fibrin network within 15 min. In a rabbit bone defect model, T-PRF membranes applied for 30 days enhanced connective tissue and bone regeneration, demonstrating the potent role of PRF-mediated clots in osseointegration.^147,149,150^

**Fig. 11:**
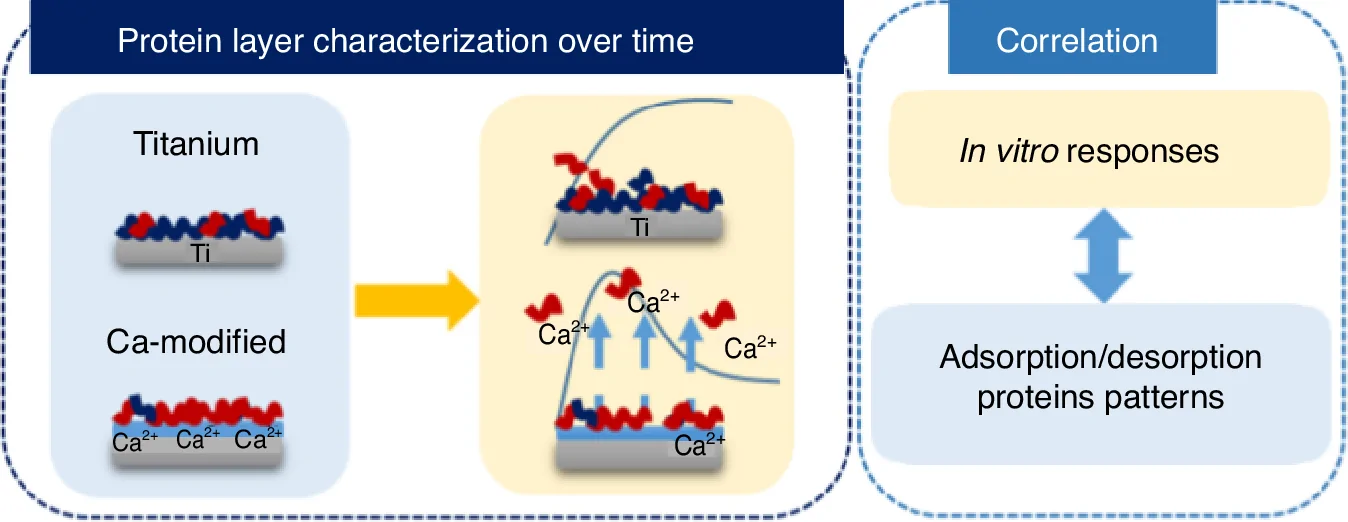
Schematic diagram of the differential biological reaction between the protein adsorption layer and the titanium surface modified by calcium ions.^151^ Copyright 2021, Romero-Gavilan, F. et al

Proteomic studies reveal that titanium surface composition governs selective protein adsorption. Romero-Gavilán et al. showed that calcium-enriched titanium selectively adsorbed 19 serum proteins, including coagulation factors (FA10, THRB, ANT3) and immune proteins such as complement C1S (2.98-fold), CO9 (2.52-fold), and immunoglobulin KV302. These adsorption patterns shaped downstream cellular responses and enhanced osteogenic differentiation (Fig. 11).^151^

However, excessive or nonspecific protein accumulation may impair cell adhesion and osteogenesis.^152^ Xu et al. addressed this by grafting a zwitterionic polymer, poly[2-(methacryloyloxy) ethyl choline phosphate] (PMCP), onto titanium via surface-initiated atom transfer radical polymerization. PMCP-coated surfaces reduced nonspecific protein adsorption by ~65%, improved hydrophilicity, and enhanced MC3T3-E1 cell adhesion and osteogenic marker expression (ALP activity increased 1.9-fold), illustrating the benefit of controlled protein–material interactions.^153^

Recent advances in self-assembling biomaterials offer additional strategies for modulating growth factor availability. Peptide amphiphile (PA)-based hydrogels co-assemble with blood components during coagulation, preserving platelet function, sustaining local release of endogenous growth factors (PDGF-AB, VEGF), and supporting in vitro proliferation of mesenchymal stromal cells, endothelial cells, and fibroblasts (the core osteogenic/angiogenic effects of which have been elaborated in Section “Cytokines in blood clot”). Autologous PA-blood gel implants successfully promoted bone regeneration in critical-sized rat calvarial defects, with new bone area reaching ~72% of the defect after 8 weeks.^105^ Collectively, these studies underscore that selective modulation of protein and growth factor adsorption and release on biomaterial surfaces directly influences cellular responses, tissue integration, and the overall success of bone regeneration strategies.

### Blood-derived materials

In addition to directly regulating clot formation through surface physicochemical modulation, blood-derived materials provide a tailored and biocompatible strategy by recapitulating key features of the native coagulation microenvironment in bone defects. Recent advances in materials science have provided effective solutions to challenges in tissue regeneration. Among them, ECM-mimetic biomaterials have received considerable attention for their ability to recapitulate key roles of the extracellular matrix (ECM) during bone healing.^6^ These bioinspired constructs replicate the hierarchical structure and mechanical properties of native tissues across multiple scales, providing topographical and physicochemical cues that guide cell proliferation, migration, and differentiation.

While synthetic ECM analogs are highly tunable, allowing properties such as stiffness, porosity, and degradation kinetics to be precisely adjusted,^154,155^ they often lack the full biological complexity of native blood-derived matrices. Conversely, autologous fibrin (a natural polymer formed by thrombin-mediated conversion of fibrinogen) has shown therapeutic potential in the repair of bone, cartilage, nerve,^156^ and vascular tissues. The physicochemical properties of fibrin hydrogels can be tailored by varying fibrinogen and thrombin concentrations,^157^ providing a degree of control over matrix architecture and regenerative performance.

In addition to providing structural support, fibrin intrinsically binds autologous growth factors, thereby amplifying osteogenic signaling.^118,158^ PRP, enriched in PDGF, TGF-β1, VEGF, and IGF-1, exemplifies this principle. Its regenerative efficacy is dose-dependent: a fivefold concentration relative to whole blood markedly enhances bone formation, whereas excessive enrichment (e.g., sixfold) exerts inhibitory or cytotoxic effects on osteoblast activity.^104,118,159^

Despite these advantages, the limited tunability of pure autologous blood-derived materials constrains their broader application. A promising strategy is therefore to integrate bioactive materials with autologous blood components. Such hybrid systems combine the biological activity of natural matrices with the adjustable features of engineered constructs, enabling improved control over clot architecture, growth factor release, and subsequent cell–material interactions.

#### Fibrin prefabricated materials

Fibrin has been widely investigated as a prefabricated matrix due to its degradability, ECM-mimicking structure, and intrinsic bioactivity. To evaluate its osteogenic potential, human umbilical cord mesenchymal stem cells (hUCMSCs) were encapsulated in alginate–fibrin microbeads (diameter: several hundred microns). Cells were efficiently released after 4 days, exhibiting robust proliferation, osteogenic differentiation, and bone mineralization. In contrast, alginate-only microbeads degraded only partially after 21 days and failed to support cell proliferation, highlighting the advantage of fibrin in generating macropores that facilitate migration and fluid circulation. Furthermore, fibrin concentration allows regulation of bead integrity and degradability, with 0.1% fibronectin identified as optimal for stable bead formation.^160^

**Fig. 12:**
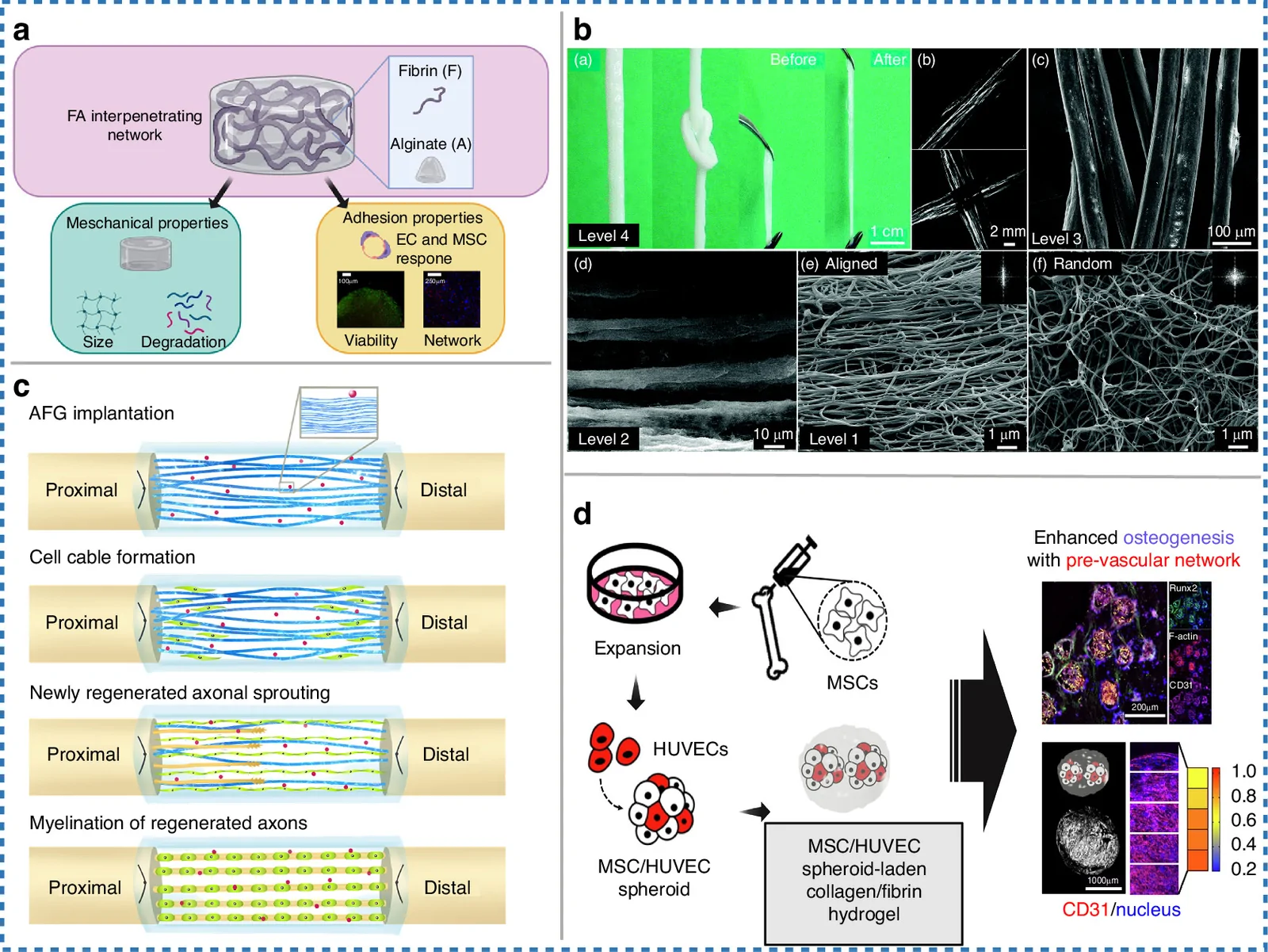
Design and biological functions of fibrin‑based hydrogels. **a** Material design of fibrin-alginate interpenetrating network (IPN) hydrogel and mechanism of the effect of tunable mechanical properties on cell adhesion^161^. Copyright 2020, Elsevier. **b** Hierarchically aligned fibrillar fibrin hydrogel fabricated by electrospinning.^165^ Copyright 2009, Royal Society of Chemistry. **c** Summarized Schematic of regenerative process occurring within AFG@CST.^166^ Copyright 2017, Elsevier. **d** Preparation of hydrogels encapsulating MSC/HUVEC spheroids and their application in providing an instructive 3D microenvironment for bone tissue formation.^178^ Copyright 2019, Elsevier

While fibrin hydrogels formed by fibrinogen polymerization are regulated, single-polymer hydrogels have limited tunability. In contrast, fibrin hydrogels formed by interpenetrating network (IPN) polymerization allow independent regulation of physicochemical properties. For example, a fibrin-alginate IPN hydrogel, developed using fibrin and sodium alginate (Fig. 12a), balances tunable mechanical properties with desirable adhesion characteristics of the material.^161^ The fibrin structure in the IPN is regulated by thrombin concentration, while the mesh size and mechanical properties are influenced by CaCl_2_ concentration. This independent tunability enables the creation of materials optimized for cell adhesion. Incorporating metal ions, particularly iron ions, has been shown to alter the mechanical strength of alginate hydrogels.^162^ To prevent cytotoxicity due to the rapid release of metal ions, metal nanoparticles can be employed as ion reservoirs. For example, incorporating iron nanoparticles into hydrogels is an effective strategy to enhance mechanical response. Alginate hydrogels containing 200 µg/mL Fe-NPs showed a 1.9-fold increase in both storage and elastic moduli.^163^ Moreover, iron ions strongly synergize with fibrin adsorption, further enhancing the cellular response under the combined effect of these materials.

Aligned fibrous fibrin hydrogels mimic the native neural ECM, whose softness is critical for designing nerve guidance conduits.^164^ Aligned fibrillar fibrin hydrogels (AFGs), fabricated via electrospinning, generate 3D aligned fibril arrays (~100 nm) that direct hUCMSC proliferation and neurite extension of dorsal root ganglion neurons (Fig. 12b).^165^ In another study, Du et al. employed a biomimetic strategy to fabricate structurally tunable AFGs and confirmed that implanted artificial fibrin hydrogels mimicked autologous nerve fibers in the early stages of nerve regeneration (Fig. 12c).^166^ These studies lay the foundation for further research into biomaterials for bone-nerve regeneration.

Beyond structural cues, fibrin has been exploited as a delivery vehicle for BMP-2, one of the most potent osteoinductive factors.^167^ Although BMP-2 induces osteogenesis in a dose-dependent manner,^168^ excessive doses (>1 μg/kg) may trigger inflammation or ectopic bone formation.^169^ Fibrin hydrogels, with their controllable degradability and ECM-like junctions, offer distinct advantages as BMP-2 delivery systems.^170^ For example, BMP-2–loaded bioactive glass microspheres combined with autologous blood clots enhanced femoral defect repair more effectively than BMP-2 injection or microspheres alone.^171^ To further enhance performance at the defect site, a controlled localized BMP-2 delivery system was developed. Previous studies have shown that fibrin glue (FG) can be used for controlled BMP delivery.^172^ Similarly, rhBMP-2-loaded gelatin/nHAp/fibrin scaffolds achieved complete recanalization of rabbit radius defects within 12 weeks.^173^ PEGylated fibrinogen hydrogels released only 7% of loaded BMP-2 over 9 days, significantly improving bone density in mouse calvarial defects.^174^ Incorporation of heparin into fibrin glue further reduced burst release, with BMP-2 release limited to 20% on day 1 versus >50% without heparin, sustaining delivery for 12 days.^169,175^

In bone trauma, enhancing tissue regeneration is critical for restoring structural integrity. Fibrin hydrogels serve as temporary ECMs that deliver growth factors and support cell proliferation.^97^ Incorporation of LM-111 (450 μg/mL) produces finer fibrin fibers with increased porosity, slows hydrogel degradation, and reduces modulus from 6 to 2 kPa, matching native muscle stiffness and promoting myocyte proliferation and growth factor secretion.^176,177^ Moreover, fibrin hydrogels can encapsulate MSC/HUVEC spheroids, providing a 3D ECM environment that improves cell spreading, osteogenic differentiation, and pre-vascular network formation, with MSCs and HUVECs acting synergistically to modulate angiogenic capacity (Fig. 12d).^178^

Additionally, the concentration of NaCl has been shown to influence the fibrin mass-to-length ratio,^179^ and thus, fibrin gels with tunable mechanical properties can be customized to replicate the ECM by adjusting the NaCl concentration in the pregel solution. Davis et al. investigated the effects of 0–4.4% (w/v) NaCl concentrations on the fibrin network and examined the resulting osteogenic properties.^180,181^ Their data revealed that when the NaCl concentration was 2.6%, the strength of the network structure reached a peak value of 17 kPa, and the alkaline phosphatase (ALP) activity also correlated with changes in NaCl concentration. The formation of hydrogels with higher fibrinogen content is known to enhance ALP activity;^162^ However, through NaCl modulation, hydrogels with tunable mechanical strength can be produced at lower salt and fibrinogen concentrations to regulate osteogenic activity.

Physical forces also play a critical role in directing stem cell fate. Zhang et al. reported a modulus of 173 Pa for MSC differentiation into an endothelial cell (EC)-like phenotype.^182^ Similarly, Mendez et al. used fibrin sealants to mimic this phenomenon.^183^ Adipose-derived stem cells (ADSCs) cultured in fibrin gels with moduli of 3 000 Pa and 1 650 Pa exhibited markedly different differentiation outcomes: ~49% of ADSCs in the 3 000 Pa gel expressed VE-cadherin after two weeks, compared to only 5% in the 1 650 Pa gel. These results highlight the importance of mechanical forces in directing stem cell differentiation and underscore the potential of fibrin-based scaffolds as carriers for stem cell delivery in tissue repair.

An appropriate inflammatory response is crucial for initiating bone tissue regeneration.^184,185^ Uncontrolled or prolonged inflammation can impair healing, whereas a properly regulated immune response promotes angiogenesis and bone repair. Thus, material design should aim to modulate the immune microenvironment to facilitate tissue regeneration. Tunable hydrogels, as ECM mimics, have shown potential in directing immune responses at injury sites.

**Fig. 13:**
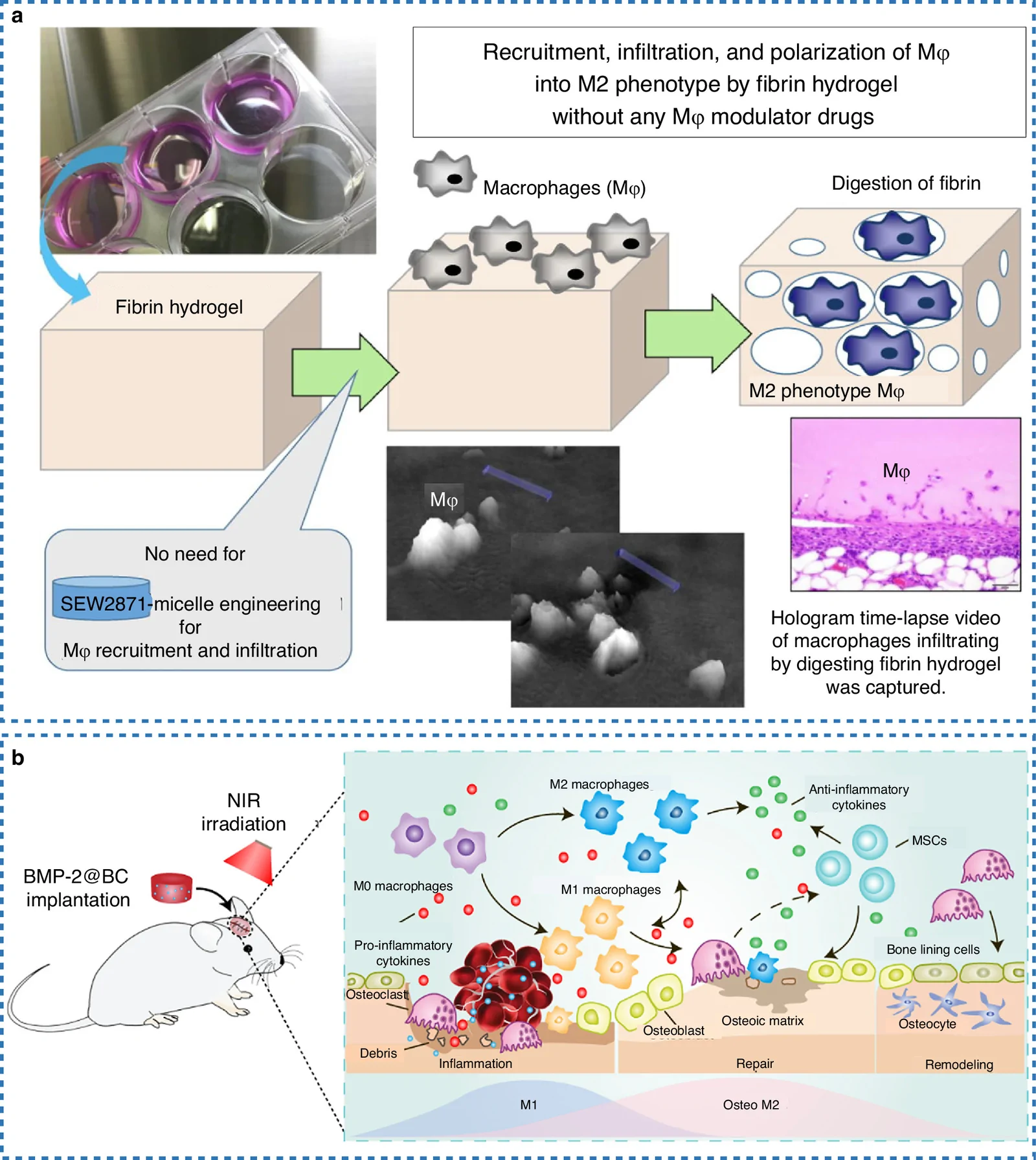
Immunomodulation and bone repair mediated by blood clot–based hydrogel. **a** Schematic representation of anti-inflammatory polarization in vivo and in vitro after combining fibrin hydrogel with macrophage recruiter (SEW2871).^186^ Copyright 2019, Elsevier. **b** Schematic representation of the effects of BMP-2-loaded hemagglutination hydrogels on bone immunity, bone repair and bone regeneration after implantation into rat cranial defects.^170^ Copyright 2021, Fan, Q et al

Fibrin hydrogels can recruit macrophages and induce anti-inflammatory polarization by adjusting fibrin content, promoting factors such as IL-10 and Fizz-1 a response not observed in gelatin hydrogels highlighting the unique immunomodulatory role of blood clot-derived matrices (Fig. 13a).^186^ Moreover, autologous blood clots can serve as responsive immunomodulatory materials; their deep red color allows near-infrared (NIR) light-mediated hyperthermia (40–43 °C) to dynamically regulate macrophage recruitment and phenotype at different healing stages. This strategy enhances pro-inflammatory signals initially to activate bone repair and subsequently promotes M2-type anti-inflammatory macrophages after 14 days, accelerating defect healing (Fig. 13b).^170^ Being autologous and non-toxic, such blood clot-based materials hold strong clinical potential for immune-guided bone regeneration.

#### Platelet-rich blood preformation materials

Building on interactions between biomaterials and autologous blood components, incorporating platelet-enriched cellular fractions further optimizes clot regulation and helps bridge early clot stabilization with downstream osteogenic differentiation. Platelets, as central regulators of coagulation and wound healing, also serve as natural reservoirs of diverse growth factors that play pivotal roles in tissue regeneration. Leveraging blood-derived materials, particularly those enriched with platelets has become a promising strategy in bone tissue engineering due to their ability to promote osteogenesis, angiogenesis, and inflammatory modulation. In this context, several studies have focused on the use of PRP and PRF as preformed components in biomaterial design to support bone repair.

A study^187^ utilized HAp microgranules polarized under a 4 kV/cm direct-current field at 400 °C for 1 h, combining them with PRP to form hydrogels. The electrically polarized ceramic particles were dispersed throughout the PRP’s fibrin network, functioning as a cellular scaffold and activating cells to exert a synergistic effect that enhanced osteogenesis.

PRF, a second-generation platelet concentrate, offers several advantages over PRP, including a simpler preparation, lack of anticoagulants, formation of a mature fibrin network, enhanced osteoblast adhesion, and upregulation of collagen expression.^188–190^ Incorporation of PRF into 3D-printed calcium phosphate scaffolds improves surface roughness and porosity, facilitating cell infiltration and distribution. SSCs cultured on BCP/PVA/PRF scaffolds exhibit elongated pseudopodia, strong adhesion, and enhanced osteogenic differentiation. In vivo, PRF-containing scaffolds accelerate defect repair, with new bone formation observed after 8 weeks, whereas PRF-free scaffolds remained surrounded by cartilage tissue.^191^

Beyond acting as clot analogs, platelet-rich blood-derived matrices also function as reservoirs for growth factors (GFs) and cytokines that orchestrate tissue repair.^192,193^ Controlled release of these factors is critical for efficacy; adsorption onto material surfaces or encapsulation within biomaterials prolongs release and prevents burst effects. For instance, chitosan–chondroitin sulfate nanoparticles adsorb platelet lysates (PL), extending release to one week, and incorporation of PL-loaded HA microparticles into calcium phosphate cement sustains PL release for up to 8 days, upregulating osteogenic markers in human adipose-derived stem cells.^194,195^ Hydrogels that mimic natural ECM offer further advantages due to tunable polymer networks; grafting HA with tyramine groups enables electrostatic capture of positively charged GFs like TGF-β1, sustaining release for up to 14 days and promoting cell proliferation.^194^

Platelet-derived growth factors collectively orchestrate bone repair: BMPs promote progenitor cell migration, proliferation, and differentiation; FGF regulates apoptosis in mature and immature osteoblasts; IGF, PDGF, and VEGF enhance osteoblast proliferation and differentiation; VEGF facilitates cartilage-to-bone conversion; and TGF-β1 stimulates proliferation of undifferentiated mesenchymal cells. Collectively, platelet-derived factors coordinate angiogenesis–osteogenesis coupling by promoting progenitor recruitment, vascular ingrowth, and osteogenic differentiation.^196–199^ Therefore, PRP- and PRF-based systems provide a multifunctional platform for creating a regenerative clot-like microenvironment to support bone repair.

Overall, biomaterial-guided clot regulation can be achieved through physicochemical interface engineering, bioinspired clot-mimetic matrices, and cell component integration. Together, these strategies provide a rational framework for converting early hemostatic clots into instructive regenerative niches that bridge rapid bleeding control with downstream osteogenesis.

## Conclusion and future directions

In summary, hemostasis and osteogenesis are tightly coupled, with the blood clot acting as the pivotal bridge. After fracture, coagulation initiates clot formation, which not only halts bleeding but also recruits immune cells and signaling molecules that orchestrate repair. Concurrently, fibrinolysis regulates clot remodeling, and its timely dissolution creates a permissive environment for new bone formation. Thus, controlled formation and degradation of clots are indispensable for coupling hemostasis with regeneration.

Despite rapid progress, several gaps and inconsistencies remain in the current body of biomaterial-clot research. First, clot stabilization is often equated with regenerative benefit; however, excessively dense or overly stiff fibrin networks may restrict cell infiltration and vascular remodeling, indicating a critical trade-off between hemostatic robustness and regenerative permissiveness. Second, the deep biological mechanisms driving clot-mediated bone repair require further systematic investigation, as direct causal evidence linking early biomaterial-blood interactions to long-term bone regeneration remains limited. Third, robust quantitative correlations linking material architecture and surface chemistry with clot formation and subsequent regenerative outcomes remain elusive, limiting the predictive and rational design of biomaterials with controllable dose effect behavior.

Future hemostatic biomaterials for critical-sized bone defects should reinforce the interface between clot and bone while maintaining a balanced fibrinolytic microenvironment. Key design principles include: (1) instantaneous blood absorption and coagulation initiation, (2) strong protein adsorption to stabilize fibrin networks, (3) biomimicry of the repair niche to ensure seamless transition from clotting to tissue regeneration, and (4) adaptive modulation for compromised clinical scenarios through targeted strategies (such as restoring growth factors and immunomodulation). Furthermore, the clinical implementation of autologous blood-based strategies demands strict ethical compliance and internationally standardized protocols to overcome regulatory barriers.

Surface engineering offers opportunities to modulate clot architecture, regulate fibrin stability, and direct its degradation kinetics. AI-assisted structural design may help identify quantitative correlations between material parameters and clot dynamics, while bioactive functionalization can further enhance osteogenic signaling without compromising hemostatic balance. In parallel, integrating precision manufacturing with emerging in situ monitoring technologies could enable real-time evaluation and iterative optimization of biomaterial performance. Ultimately, advancing the mechanistic insights into the clot bone interface will guide the development of next-generation hemostatic biomaterials that integrate hemostasis and regeneration for robust bone repair.

## References

[1] Spahn, D. R. et al. The European guideline on management of major bleeding and coagulopathy following trauma: fifth edition. *Crit. Care* **23**, 98 (2019). 10.1186/s13054-019-2347-3 PMC6436241 30917843

[2] Naghavi, M. et al. Global, regional, and national age-sex specific mortality for 264 causes of death, 1980–2016: a systematic analysis for the Global Burden of Disease Study 2016. *Lancet* **390**, 1151–1210 (2017). 10.1016/S0140-6736(17)32152-9 PMC5605883 28919116

[3] Yang, Y. & Xiao, Y. Biomaterials regulating bone hematoma for osteogenesis. *Adv. Health. Mater.* **9**, 2000726 (2020). 10.1002/adhm.202000726 32691989

[4] Chen, S. et al. Biomaterials with structural hierarchy and controlled 3D nanotopography guide endogenous bone regeneration. *Sci. Adv.* **7**, eabg3089 (2021). 10.1126/sciadv.abg3089 PMC8318363 34321208

[5] Chen, M. et al. Skeleton-vasculature chain reaction: a novel insight into the mystery of homeostasis. *Bone Res.* **9**, 21 (2021). 10.1038/s41413-021-00138-0 PMC7985324 33753717

[6] Zhu, G. et al. Bone physiological microenvironment and healing mechanism: Basis for future bone-tissue engineering scaffolds. *Bioact. Mater.* **6**, 4110–4140 (2021). 10.1016/j.bioactmat.2021.03.043 PMC8091181 33997497

[7] Shiu, H. T., Leung, P. C. & Ko, C. H. The roles of cellular and molecular components of a hematoma at early stage of bone healing. *J. Tissue Eng. Regener. Med.* **12**, E1911–E1925 (2018). 10.1002/term.2622 29207216

[8] Bastian, O. W., Koenderman, L., Alblas, J., Leenen, L. P. & Blokhuis, T. J. Neutrophils contribute to fracture healing by synthesizing fibronectin+ extracellular matrix rapidly after injury. *Clin. Immunol.* **164**, 78–84 (2016). 10.1016/j.clim.2016.02.001 26854617

[9] Stegen, S., van Gastel, N. & Carmeliet, G. Bringing new life to damaged bone: the importance of angiogenesis in bone repair and regeneration. *Bone* **70**, 19–27 (2015). 10.1016/j.bone.2014.09.017 25263520

[10] Adekambi, T. et al. Biomarkers on patient T cells diagnose active tuberculosis and monitor treatment response. *J. Clin. Investig.* **125**, 1827–1838 (2015). 10.1172/JCI77990 PMC4598074 25822019

[11] Kolar, P., Gaber, T., Perka, C., Duda, G. N. & Buttgereit, F. Human early fracture hematoma is characterized by inflammation and hypoxia. *Clin. Orthop. Relat. Res.* **469**, 3118–3126 (2011). 10.1007/s11999-011-1865-3 PMC3183184 21409457

[12] Wang, X., Zhang, Y., Ji, W. & Ao, J. Categorising bone defect hematomas—enhance early bone healing. *Med. Hypotheses* **113**, 77–80 (2018). 10.1016/j.mehy.2018.02.029 29523301

[13] Wu, L., Liu, J., Qiu, T. & Dai, H. The natural reticular hematoma scaffold modulating inflammatory microenvironment and promoting bone regeneration. *Compos. Part B Eng.* **295**, 112199 (2025).

[14] Walters, G., Pountos, I. & Giannoudis, P. V. The cytokines and micro-environment of fracture haematoma: current evidence. *J. Tissue Eng. Regener. Med.* **12**, e1662–e1677 (2018). 10.1002/term.2593 29047220

[15] Huangfu, Y. et al. An off-the-shelf artificial blood clot hydrogel neutralizing multiple proinflammatory mediators for pro-regenerative periodontitis treatment. *Adv. Sci.* **12**, e04106 (2025). 10.1002/advs.202504106 PMC12376679 40433947

[16] Harwood, P. J., Newman, J. B., Michael, A. L. J. O. & Trauma (ii) An update on fracture healing and non-union. *Orthop. Trauma* **28**, 228–242 (2010).

[17] Woloszyk, A. et al. Fracture hematoma micro-architecture influences transcriptional profile and plays a crucial role in determining bone healing outcomes. *Biomater. Adv.* **139**, 213027 (2022). 10.1016/j.bioadv.2022.213027 35882120

[18] Echeverri, L. F., Herrero, M. A., Lopez, J. M. & Oleaga, G. Early stages of bone fracture healing: formation of a fibrin-collagen scaffold in the fracture hematoma. *Bull. Math. Biol.* **77**, 156–183 (2015). 10.1007/s11538-014-0055-3 25537828

[19] Shiu, H. T., Goss, B., Lutton, C., Crawford, R. & Xiao, Y. Formation of Blood Clot on Biomaterial Implants Influences Bone Healing. *Tissue Eng. Part B-Rev.* **20**, 697–712 (2014). 10.1089/ten.TEB.2013.0709 24906469

[20] Sung, Y. K., Lee, D. R. & Chung, D. J. Advances in the development of hemostatic biomaterials for medical application. *Biomater. Res* **25**, 37 (2021). 10.1186/s40824-021-00239-1 PMC8588689 34772454

[21] Diethorn, M. L. & Weld, L. M. Physiologic mechanisms of hemostasis and fibrinolysis. *J. Cardiovasc. Nurs.* **4**, 1–10 (1989). 10.1097/00005082-198911000-00002 2689600

[22] Khonsary, S. Guyton and Hall: textbook of medical physiology. *Surg. Neurol. Int.* **8**, 275 (2017).

[23] Rand, M. L. et al. Phosphatidylserine exposure and other apoptotic-like events in Bernard-Soulier syndrome platelets. *Am. J. Hematol.* **85**, 584–592 (2010). 10.1002/ajh.21768 20658588

[24] Nurden, A. T. Platelets, inflammation and tissue regeneration. *Thromb. Haemost.* **105**, S13–S33 (2011). 10.1160/THS10-11-0720 21479340

[25] Wang, S. et al. Current applications of platelet gels in wound healing—a review. *Wound Repair Regen.* **29**, 370–379 (2021). 10.1111/wrr.12896 33749992

[26] Heemskerk, J. W., Bevers, E. M. & Lindhout, T. Platelet activation and blood coagulation. *Thromb. Haemost.* **88**, 186–193 (2002). 12195687

[27] Versteeg, H. H., Heemskerk, J. W., Levi, M. & Reitsma, P. H. New fundamentals in hemostasis. *Physiol. Rev.* **93**, 327–358 (2013). 10.1152/physrev.00016.2011 23303912

[28] Harper, D., Young, A. & McNaught, C.-E. The physiology of wound healing. *Surg. - Oxf. Int. Ed.* **32**, 445–450 (2014).

[29] Kilian, O. et al. New blood vessel formation and expression of VEGF receptors after implantation of platelet growth factor-enriched biodegradable nanocrystalline hydroxyapatite. *Growth Factors* **23**, 125–133 (2005). 10.1080/08977190500126306 16019434

[30] Lord, S. T. Fibrinogen and fibrin: scaffold proteins in hemostasis. *Curr. Opin. Hematol.* **14**, 236–241 (2007). 10.1097/MOH.0b013e3280dce58c 17414213

[31] Behrens, A. M., Sikorski, M. J. & Kofinas, P. Hemostatic strategies for traumatic and surgical bleeding. *J. Biomed. Mater. Res A* **102**, 4182–4194 (2014). 10.1002/jbm.a.35052 PMC5663245 24307256

[32] Wang, X. et al. Alteration of clot architecture using bone substitute biomaterials (beta-tricalcium phosphate) significantly delays the early bone healing process. *J. Mater. Chem. B*. **6** (2018). 10.1039/c8tb01747f 32254940

[33] Girard, N., Cauvin, E. R. J., Gauthier, O. & Gault, S. Biphasic calcium phosphate microparticles mixed with autologous blood: application for the reconstruction of a large mandibular bone defect in a dog. *J. Vet. Dent.* **37**, 201–209 (2020). 10.1177/0898756421990909 33601942

[34] Yuasa, M. et al. Fibrinolysis is essential for fracture repair and prevention of heterotopic ossification. *J. Clin. Investig.* **125**, 3117–3131 (2015). 10.1172/JCI80313 PMC4563750 26214526

[35] Karp, J. M., Sarraf, F., Shoichet, M. S. & Davies, J. E. Fibrin-filled scaffolds for bone-tissue engineering: an in vivo study. *J. Biomed. Mater. Res A* **71**, 162–171 (2004). 10.1002/jbm.a.30147 15368266

[36] Michael, C. et al. Combined computational modeling and experimental study of the biomechanical mechanisms of platelet-driven contraction of fibrin clots. *Commun. Biol.* **6**, 869 (2023). 10.1038/s42003-023-05240-z PMC10449797 37620422

[37] Wolberg, A. S. & Campbell, R. A. Thrombin generation, fibrin clot formation and hemostasis. *Transfus. Apher. Sci.* **38**, 15–23 (2008). 10.1016/j.transci.2007.12.005 PMC2408736 18282807

[38] Wolberg, A. S. Thrombin generation and fibrin clot structure. *Blood Rev.* **21**, 131–142 (2007). 10.1016/j.blre.2006.11.001 17208341

[39] Bennett, J. S., Berger, B. W. & Billings, P. C. The structure and function of platelet integrins. *J. Thromb. Haemost.* **7**, 200–205 (2009). 10.1111/j.1538-7836.2009.03378.x 19630800

[40] Raghavendran, H. R. B. et al. Synergistic interaction of platelet-derived growth factor (PDGF) with the surface of PLLA/Col/HA and PLLA/HA scaffolds produces rapid osteogenic differentiation. *Colloids Surf. B-Biointerfaces* **139**, 68–78 (2016). 10.1016/j.colsurfb.2015.11.053 26700235

[41] Fan, W., Crawford, R. & Xiao, Y. The ratio of VEGF/PEDF expression in bone marrow mesenchymal stem cells regulates neovascularization. *Differentiation* **81**, 181–191 (2011). 10.1016/j.diff.2010.12.003 21236558

[42] Kells, A. F., Coats, S. R., Schwartz, H. S. & Hoover, R. L. TGF-β and PDGF act synergistically in affecting the growth of human osteoblast-enriched cultures. *Connect. Tissue Res.* **31**, 117–124 (1995). 10.3109/03008209509028399 15612327

[43] Solovieva, A. et al. Immobilization of platelet-rich plasma onto COOH plasma-coated PCL nanofibers boost viability and proliferation of human mesenchymal stem cells. *Polymers*. **9** (2017). 10.3390/polym9120736 PMC6418517 30966035

[44] Kolar, P. et al. The early fracture hematoma and its potential role in fracture healing. *Tissue Eng. Part B Rev.* **16**, 427–434 (2010). 10.1089/ten.TEB.2009.0687 20196645

[45] Bab, I. et al. Kinetics and differentiation of marrow stromal cells in diffusion chambers in vivo. *J. Cell Sci.* **84**, 139–151 (1986). 10.1242/jcs.84.1.139 3805151

[46] Cho, T.-J., Gerstenfeld, L. C. & Einhorn, T. A. Differential temporal expression of members of the transforming growth factor β superfamily during murine fracture healing. *J. Bone Miner. Res.* **17**, 513–520 (2002). 10.1359/jbmr.2002.17.3.513 11874242

[47] Kon, T. et al. Expression of osteoprotegerin, receptor activator of NF-κB ligand (osteoprotegerin ligand) and related proinflammatory cytokines during fracture healing. *J. Bone Miner. Res.* **16**, 1004–1014 (2001). 10.1359/jbmr.2001.16.6.1004 11393777

[48] Li, L. et al. Sequential gastrodin release PU/n-HA composite scaffolds reprogram macrophages for improved osteogenesis and angiogenesis. *Bioact. Mater.* **19**, 24–37 (2023). 10.1016/j.bioactmat.2022.03.037 PMC8980440 35415312

[49] Pieters, M. & Wolberg, A. S. Fibrinogen and fibrin: an illustrated review. *Res. Pract. Thromb. Haemost.* **3**, 161–172 (2019). 10.1002/rth2.12191 PMC6462751 31011700

[50] Klatt, C. et al. Platelet-RBC interaction mediated by FasL/FasR induces procoagulant activity important for thrombosis. *J. Clin. Investig.* **128**, 3906–3925 (2018). 10.1172/JCI92077 PMC6118585 29952767

[51] Weisel, J. W. & Litvinov, R. I. Red blood cells: the forgotten player in hemostasis and thrombosis. *J. Thromb. Haemost.* **17**, 271–282 (2019). 10.1111/jth.14360 PMC6932746 30618125

[52] Diaz-Rodriguez, P., Gonzalez, P., Serra, J. & Landin, M. Key parameters in blood-surface interactions of 3D bioinspired ceramic materials. *Mater. Sci. Eng. C-Mater. Biol. Appl.* **41**, 232–239 (2014). 10.1016/j.msec.2014.04.058 24907756

[53] Sperling, C., Fischer, M., Maitz, M. F. & Werner, C. Blood coagulation on biomaterials requires the combination of distinct activation processes. *Biomaterials* **30**, 4447–4456 (2009). 10.1016/j.biomaterials.2009.05.044 19535136

[54] Ma, W. J. et al. DLC coatings: effects of physical and chemical properties on biological response. *Biomaterials* **28**, 1620–1628 (2007). 10.1016/j.biomaterials.2006.12.010 17196649

[55] Huang, H.-H. et al. Blood responses to titanium surface with TiO_2_ nano-mesh structure. *Clin. Oral. Implants Res.* **23**, 379–383 (2012). 10.1111/j.1600-0501.2010.02152.x 21457350

[56] Quan, K. et al. Black hemostatic sponge based on facile prepared cross-linked graphene. *Colloids Surf. B Biointerfaces* **132**, 27–33 (2015). 10.1016/j.colsurfb.2015.04.067 26001799

[57] Zhang, W. et al. Paraffin-coated hydrophobic hemostatic zeolite gauze for rapid coagulation with minimal adhesion. *ACS Appl. Mater. Interfaces* **13**, 52174–52180 (2021). 10.1021/acsami.1c10891 34554720

[58] Park, J.-Y., Kyung, K.-H., Tsukada, K., Kim, S.-H. & Shiratori, S. Biodegradable polycaprolactone nanofibres with β-chitosan and calcium carbonate produce a hemostatic effect. *Polymer* **123**, 194–202 (2017).

[59] Zhu, T. et al. Superhydrophobic/superhydrophilic Janus fabrics reducing blood loss. *Adv. Health. Mater.* **7**, e1701086 (2018). 10.1002/adhm.201701086 29334429

[60] He, M. et al. Bioinspired interfacial engineering of multiscale micro/nano-structured surfaces with robust superhydrophobic and tunable adhesion. *Chem. Eng. J.* **501**, 157482 (2024).

[61] Li, Z. et al. Superhydrophobic hemostatic nanofiber composites for fast clotting and minimal adhesion. *Nat. Commun.* **10**, 11 (2019). 10.1038/s41467-019-13512-8 PMC6895059 31804481

[62] Ekdahl, K. N. et al. Innate immunity activation on biomaterial surfaces: a mechanistic model and coping strategies. *Adv. Drug Deliv. Rev.* **63**, 1042–1050 (2011). 10.1016/j.addr.2011.06.012 PMC3166435 21771620

[63] Qi, J., Zhang, H., Wang, Y., Mani, M. P. & Jaganathan, S. K. Development and blood compatibility assessment of electrospun polyvinyl alcohol blended with metallocene polyethylene and plectranthus amboinicus (PVA/mPE/PA) for bone tissue engineering. *Int. J. Nanomed.* **13**, 2777–2788 (2018). 10.2147/IJN.S151242 PMC5955049 29785105

[64] Huang, N. et al. Hemocompatibility of titanium oxide films. *Biomaterials* **24**, 2177–2187 (2003). 10.1016/s0142-9612(03)00046-2 12699653

[65] Ricklin, D. & Lambris, J. D. Progress and trends in complement therapeutics. *Adv. Exp. Med. Biol.* **735**, 1–22 (2013). 10.1007/978-1-4614-4118-2_1 PMC3526805 22990692

[66] Anderson, J. M., Rodriguez, A. & Chang, D. T. Foreign body reaction to biomaterials. *Semin Immunol.* **20**, 86–100 (2008). 10.1016/j.smim.2007.11.004 PMC2327202 18162407

[67] Yu, L. et al. Highly efficient artificial blood coagulation shortcut confined on Ca-zeolite surface. *Nano Res.* **14**, 3309–3318 (2021).

[68] Sperling, C., Schweiss, R. B., Streller, U. & Werner, C. In vitro hemocompatibility of self-assembled monolayers displaying various functional groups. *Biomaterials* **26**, 6547–6557 (2005). 10.1016/j.biomaterials.2005.04.042 15939466

[69] Tan, J. S., Butterfield, D. E., Voycheck, C. L., Caldwell, K. D. & Li, J. T. Surface modification of nanoparticles by PEO/PPO block copolymers to minimize interactions with blood components and prolong blood circulation in rats. *Biomaterials* **14**, 823–833 (1993). 10.1016/0142-9612(93)90004-l 8218736

[70] Kim, D., El-Shall, H., Dennis, D. & Morey, T. Interaction of PLGA nanoparticles with human blood constituents. *Colloids Surf. B-Biointerfaces* **40**, 83–91 (2005). 10.1016/j.colsurfb.2004.05.007 15642458

[71] Cho, J. & Mosher, D. F. Enhancement of thrombogenesis by plasma fibronectin cross-linked to fibrin and assembled in platelet thrombi. *Blood* **107**, 3555–3563 (2006). 10.1182/blood-2005-10-4168 PMC1457097 16391013

[72] Mosher, D. F. Cross-linking of cold-insoluble globulin by fibrin-stabilizing factor. *J. Biol. Chem.* **250**, 6614–6621 (1975). 1158872

[73] Gossart, A. et al. Coating of cobalt chrome substrates with thin films of polar/hydrophobic/ionic polyurethanes: characterization and interaction with human immunoglobulin G and fibronectin. *Colloids Surf. B-Biointerfaces* **179**, 114–120 (2019). 10.1016/j.colsurfb.2019.03.040 30952017

[74] Shang, X. et al. Unusual zymogen activation patterns in the protein corona of Ca-zeolites. *Nat. Catal.* **4**, 607 (2021).

[75] Walsh, T. G., Metharom, P. & Berndt, M. C. The functional role of platelets in the regulation of angiogenesis. *Platelets* **26**, 199–211 (2015). 10.3109/09537104.2014.909022 24832135

[76] Huang, J. et al. Platelet integrin alphaIIbbeta3: signal transduction, regulation, and its therapeutic targeting. *J. Hematol. Oncol.* **12**, 26 (2019). 10.1186/s13045-019-0709-6 PMC6407232 30845955

[77] Sekhon, U. D. S. & Sen Gupta, A. Platelets and platelet-inspired biomaterials technologies in wound healing applications. *Acs Biomater. Sci. Eng.* **4**, 1176–1192 (2018). 10.1021/acsbiomaterials.7b00013 33418655

[78] Ruggeri, Z. M. & Mendolicchio, G. L. Adhesion mechanisms in platelet function. *Circ. Res* **100**, 1673–1685 (2007). 10.1161/01.RES.0000267878.97021.ab 17585075

[79] Hickman, D. A., Pawlowski, C. L., Sekhon, U. D. S., Marks, J. & Gupta, A. S. Biomaterials and advanced technologies for hemostatic management of bleeding. *Adv. Mater.* **30**, 201700859 (2018). 10.1002/adma.201700859 PMC5831165 29164804

[80] Zhai, Z. et al. Co-assembled supramolecular hydrogels of cell adhesive peptide and alginate for rapid hemostasis and efficacious wound healing. *Soft Matter* **15**, 8603–8610 (2019). 10.1039/c9sm01296f 31616890

[81] Lee, K. Y. & Mooney, D. J. Alginate: properties and biomedical applications. *Prog. Polym. Sci.* **37**, 106–126 (2012). 10.1016/j.progpolymsci.2011.06.003 PMC3223967 22125349

[82] Rhee, P. et al. QuikClot use in trauma for hemorrhage control: case series of 103 documented uses. *J. Trauma Acute Care Surg*. **64** (2008). 10.1097/TA.0b013e31812f6dbc 18404080

[83] Davies, J. E. Understanding peri-implant endosseous healing. *J. Dent. Educ.* **67**, 932–949 (2003). 12959168

[84] Kikuchi, L., Park, J. Y., Victor, C. & Davies, J. E. Platelet interactions with calcium-phosphate-coated surfaces. *Biomaterials* **26**, 5285–5295 (2005). 10.1016/j.biomaterials.2005.01.009 15814126

[85] Lam, W. A. et al. Mechanics and contraction dynamics of single platelets and implications for clot stiffening. *Nat. Mater.* **10**, 61–66 (2011). 10.1038/nmat2903 PMC3236662 21131961

[86] Bandyopadhyay, D., Baruah, H., Gupta, B. & Sharma, S. Silver nano particles prevent platelet adhesion on immobilized fibrinogen. *Indian J. Clin. Biochem.* **27**, 164–170 (2012). 10.1007/s12291-011-0169-4 PMC3358372 23543820

[87] Shrivastava, S. et al. Characterization of antiplatelet properties of silver nanoparticles. *ACS Nano* **3**, 1357–1364 (2009). 10.1021/nn900277t 19545167

[88] Tsuji, K. et al. BMP2 activity, although dispensable for bone formation, is required for the initiation of fracture healing. *Nat. Genet.* **38**, 1424–1429 (2006). 10.1038/ng1916 17099713

[89] Wang, X., Friis, T., Glatt, V., Crawford, R. & Xiao, Y. Structural properties of fracture haematoma: current status and future clinical implications. *J. Tissue Eng. Regener. Med.* **11**, 2864–2875 (2017). 10.1002/term.2190 27401283

[90] Pomini, K. T. et al. Fibrin sealant derived from human plasma as a scaffold for bone grafts associated with photobiomodulation therapy. *Int. J. Mol. Sci.* **20**, 1761 (2019). 10.3390/ijms20071761 PMC6479442 30974743

[91] Groothuis, A. et al. Mechanical stimulation of the pro-angiogenic capacity of human fracture haematoma: involvement of VEGF mechano-regulation. *Bone* **47**, 438–444 (2010). 10.1016/j.bone.2010.05.026 20580871

[92] Schindeler, A., McDonald, M. M., Bokko, P. & Little, D. G. Bone remodeling during fracture repair: the cellular picture. *Semin Cell Dev. Biol.* **19**, 459–466 (2008). 10.1016/j.semcdb.2008.07.004 18692584

[93] Yu, X., Tang, X., Gohil, S. V. & Laurencin, C. T. Biomaterials for bone regenerative engineering. *Adv. Health. Mater.* **4**, 1268–1285 (2015). 10.1002/adhm.201400760 PMC4507442 25846250

[94] Shats, E. A., Nair, C. H. & Dhall, D. P. Interaction of endothelial cells and fibroblasts with modified fibrin networks: role in atherosclerosis. *Atherosclerosis* **129**, 9–15 (1997). 10.1016/s0021-9150(96)06003-0 9069511

[95] Chen, Y., Li, W., Zhang, C., Wu, Z. & Liu, J. Recent developments of biomaterials for additive manufacturing of bone scaffolds. *Adv. Health. Mater.* **23**, e2000724 (2020). 10.1002/adhm.202000724 32743960

[96] Leghissa, G. C., Zaffe, D., Assenza, B. & Botticelli, A. R. Guided bone regeneration using titanium grids: report of 10 cases. *Clin. Oral. Implants Res.* **10**, 62–68 (1999). 10.1034/j.1600-0501.1999.100108.x 10196791

[97] Di Iorio, D. et al. Quantitative evaluation of the fibrin clot extension on different implant surfaces: an in vitro study. *J. Biomed. Mater. Res. Part B-Appl. Biomater.* **74B**, 636–642 (2005). 10.1002/jbm.b.30251 15803487

[98] Boix, M. et al. ATR-FTIR measurements of albumin and fibrinogen adsorption: Inert versus calcium phosphate ceramics. *J. Biomed. Mater. Res. Part A* **103**, 3493–3502 (2015). 10.1002/jbm.a.35496 25940865

[99] Shakir, M., Jolly, R., Khan, M. S., Rauf, A. & Kazmi, S. Nano-hydroxyapatite/beta-CD/chitosan nanocomposite for potential applications in bone tissue engineering. *Int. J. Biol. Macromol.* **93**, 276–289 (2016). 10.1016/j.ijbiomac.2016.08.046 27543347

[100] Fernandez-Montes Moraleda, B., San Roman, J. & Rodriguez-Lorenzo, L. M. Influence of surface features of hydroxyapatite on the adsorption of proteins relevant to bone regeneration. *J. Biomed. Mater. Res. Part A* **101**, 2332–2339 (2013). 10.1002/jbm.a.34528 23359512

[101] Rosengren, Å et al. Plasma protein adsorption pattern on characterized ceramic biomaterials. *Biomaterials* **23**, 1237–1247 (2002). 10.1016/s0142-9612(01)00244-7 11791928

[102] Young, B. R., Lambrecht, L. K., Cooper, S. L. & Mosher, D. F. Biomaterials: interfacial phenomena and applications. *Adv. Chem.* **22**, 317–350 (1982). American chemical society.

[103] Takabatake, K. et al. Effect of geometry and microstructure of honeycomb TCP scaffolds on bone regeneration. *J. Biomed. Mater. Res A* **102**, 2952–2960 (2014). 10.1002/jbm.a.34966 24115688

[104] Marx, R. E. Platelet-rich plasma (PRP): what is PRP and what is not PRP? *Implant Dent.* **10**, 225–228 (2001). 10.1097/00008505-200110000-00002 11813662

[105] Padilla-Lopategui, S. et al. Biocooperative regenerative materials by harnessing blood-clotting and peptide self-assembly. *Adv. Mater.* **36**, e2407156 (2024). 10.1002/adma.202407156 PMC11681309 39543808

[106] Preininger, B. et al. An experimental setup to evaluate innovative therapy options for the enhancement of bone healing using BMP as a benchmark—a pilot study. *Eur. Cell Mater.* **23**, 262–271 (2012). discussion 271-262. 10.22203/ecm.v023a20 22492018

[107] Milillo, L., Cinone, F., Lo Presti, F., Lauritano, D. & Petruzzi, M. The role of blood clot in guided bone regeneration: biological considerations and clinical applications with titanium foil. *Materials* **14** (2021). 10.3390/ma14216642 PMC8587813 34772167

[108] Kopf, B. S., Ruch, S., Berner, S., Spencer, N. D. & Maniura-Weber, K. The role of nanostructures and hydrophilicity in osseointegration: in-vitro protein-adsorption and blood-interaction studies. *J. Biomed. Mater. Res. Part A* **103**, 2661–2672 (2015). 10.1002/jbm.a.35401 25631679

[109] Alexandre Gehrke, S. et al. Effect of different morphology of titanium surface on the bone healing in defects filled only with blood clot: a new animal study design. *Biomed. Res. Int.* **2018**, 4265474 (2018). 10.1155/2018/4265474 PMC6106843 30175131

[110] Yang, J., Zhou, Y., Wei, F. & Xiao, Y. Blood clot formed on rough titanium surface induces early cell recruitment. *Clin. Oral. Implants Res.* **27**, 1031–1038 (2016). 10.1111/clr.12672 26332946

[111] Weiner, S., Simon, J., Ehrenberg, D. S., Zweig, B. & Ricci, J. L. The effects of laser microtextured collars upon crestal bone levels of dental implants. *Implant Dent.* **17**, 217–228 (2008). 10.1097/ID.0b013e3181779016 18545054

[112] MacDonald, D. E., Deo, N., Markovic, B., Stranick, M. & Somasundaran, P. Adsorption and dissolution behavior of human plasma fibronectin on thermally and chemically modified titanium dioxide particles. *Biomaterials* **23**, 1269–1279 (2002). 10.1016/s0142-9612(01)00317-9 11791930

[113] Trento, G. D. S. et al. Bone tissue formation around two titanium implant surfaces placed in bone defects filled with bone substitute material or blood clot: a pilot study. *Clin. Implant Dent. Relat. Res* **21**, 1175–1180 (2019). 10.1111/cid.12855 31691471

[114] Bai, L. et al. Targeting early healing phase with titania nanotube arrays on tunable diameters to accelerate bone regeneration and osseointegration. *Small* **17**, e2006287 (2021). 10.1002/smll.202006287 33377275

[115] Bai, L., Chen, P., Tang, B., Hang, R. & Xiao, Y. Correlation between LncRNA profiles in the blood clot formed on nano-scaled implant surfaces and osseointegration. *Nanomaterials*. **11** (2021). 10.3390/nano11030674 PMC8001142 33803187

[116] Bai, L. et al. A micro/nano-biomimetic coating on titanium orchestrates osteo/angio-genesis and osteoimmunomodulation for advanced osseointegration. *Biomaterials* **278**, 121162 (2021). 10.1016/j.biomaterials.2021.121162 34628191

[117] Wei, F. et al. Blood prefabricated hydroxyapatite/tricalcium phosphate induces ectopic vascularized bone formation via modulating the osteoimmune environment. *Biomater. Sci.* **6**, 2156–2171 (2018). 10.1039/c8bm00287h 29931022

[118] Decco, O., Cura, A., Beltran, V., Lezcano, M. & Engelke, W. Bone augmentation in rabbit tibia using microfixed cobalt-chromium membranes with whole blood, tricalcium phosphate and bone marrow cells. *Int. J. Clin. Exp. Med.* **8**, 135–144 (2015). PMC4358437 25784982

[119] Balaguer, T. et al. Combination of blood and biphasic calcium phosphate microparticles for the reconstruction of large bone defects in dog: a pilot study. *J. Biomed. Mater. Res. Part A* **106**, 1842–1850 (2018). 10.1002/jbm.a.36384 29573560

[120] Balaguer, T. et al. Biphasic calcium phosphate microparticles for bone formation: benefits of combination with blood clot. *Tissue Eng. Part A* **16**, 3495–3505 (2010). 10.1089/ten.TEA.2010.0227 20590522

[121] Kim, B.-S. & Lee, J. Enhanced bone healing by improved fibrin-clot formation via fibrinogen adsorption on biphasic calcium phosphate granules. *Clin. Oral Implants Res*. **26** (2015). 10.1111/clr.12431 24888232

[122] Liu, R. et al. Blood prefabrication subcutaneous small animal model for the evaluation of bone substitute materials. *ACS Biomater. Sci. Eng.* **4**, 2516–2527 (2018). 10.1021/acsbiomaterials.8b00323 33435115

[123] Jing, L. et al. Proteomic analysis identified LBP and CD14 as key proteins in blood/biphasic calcium phosphate microparticle interactions. *Acta Biomater.* **127**, 298–312 (2021). 10.1016/j.actbio.2021.03.070 33831568

[124] Ko, C. L. et al. Properties of osteoconductive biomaterials: calcium phosphate cement with different ratios of platelet-rich plasma as identifiers. *Mater. Sci. Eng. C. Mater. Biol. Appl* **33**, 3537–3544 (2013). 10.1016/j.msec.2013.04.042 23706244

[125] Mellier, C. et al. A straightforward approach to enhance the textural, mechanical and biological properties of injectable calcium phosphate apatitic cements (CPCs): CPC/blood composites, a comprehensive study. *Acta Biomater.* **62**, 328–339 (2017). 10.1016/j.actbio.2017.08.040 28864250

[126] Lu, H. H., Pollack, S. R. & Ducheyne, P. 45S5 Bioactive glass surface charge variations and the formation of a surface calcium phosphate layer in a solution containing fibronectin. *J. Biomed. Mater. Res.* **54**, 454–461 (2001). 10.1002/1097-4636(20010305)54:3<454::aid-jbm200>3.0.co;2-h 11189054

[127] Putra, N. E., Mirzaali, M. J., Apachitei, I., Zhou, J. & Zadpoor, A. A. Multi-material additive manufacturing technologies for Ti-, Mg-, and Fe-based biomaterials for bone substitution. *Acta Biomater.* **109**, 1–20 (2020). 10.1016/j.actbio.2020.03.037 32268239

[128] GonzÁLez-Carrasco, J. L. Metals as bone repair materials. *Bone Repair Biomater.* **109**, 154–193 (2009).

[129] Gruenewald, T. A. et al. Reaction of bone nanostructure to a biodegrading magnesium WZ21 implant—a scanning small-angle X-ray scattering time study. *Acta Biomater.* **31**, 448–457 (2016). 10.1016/j.actbio.2015.11.049 26621693

[130] Jaiswal, S., Dubey, A., Haldar, S., Roy, P. & Lahiri, D. Differential in vitro degradation and protein adhesion behaviour of spark plasma sintering fabricated magnesium-based temporary orthopaedic implant in serum and simulated body fluid. *Biomed. Mater.* **15**, 015006 (2020). 10.1088/1748-605X/ab4f8b 31634879

[131] Tang, Y. et al. How surface electronegativity and calcium release in enamel mediate the adsorption and lubrication of salivary proteins: the role of interfacial water. *Friction* **13**, 9440912 (2025).

[132] Wang, H. et al. Effects of alloy elements on adsorption of fibrinogen on biodegradable magnesium alloys surfaces: the MD simulations and experimental studies. *Appl. Surface Sci*. **512**, 145725 (2020).

[133] Visalakshan, R. M. et al. Nanotopography-induced unfolding of fibrinogen modulates leukocyte binding and activation. *Adv. Funct. Mater.* **29** (2019).

[134] Lin, S., Van den Bergh, W., Baker, S. & Jones, J. R. Protein interactions with nanoporous sol-gel derived bioactive glasses. *Acta Biomater.* **7**, 3606–3615 (2011). 10.1016/j.actbio.2011.06.042 21757036

[135] Wang, X. Y., Xie, W. M., Zhang, D. & He, Q. Wave and vegetation effects on flow and suspended sediment characteristics: a flume study. *Estuar., Coast. Shelf Sci.* **182**, 1–11 (2016).

[136] Baker, C. J., Smith, S. A. & Morrissey, J. H. Polyphosphate in thrombosis, hemostasis, and inflammation. *Res Pract. Thromb. Haemost.* **3**, 18–25 (2019). 10.1002/rth2.12162 PMC6332810 30656272

[137] Gu, J.-T. et al. Polyphosphate-crosslinked collagen scaffolds for hemostasis and alveolar bone regeneration after tooth extraction. *Bioact. Mater.* **15**, 68–81 (2022). 10.1016/j.bioactmat.2021.12.019 PMC8940764 35386354

[138] Rother, S. et al. Sulfated hyaluronan alters endothelial cell activation in vitro by controlling the biological activity of the angiogenic factors vascular endothelial growth factor-A and tissue inhibitor of metalloproteinase-3. *ACS Appl. Mater. Interfaces* **9**, 9539–9550 (2017). 10.1021/acsami.7b01300 28248081

[139] Al-Maawi, S. et al. Covalent linkage of sulfated hyaluronan to the collagen scaffold Mucograft (R) enhances scaffold stability and reduces proinflammatory macrophage activation in vivo. *Bioact. Mater.* **8**, 420–434 (2022). 10.1016/j.bioactmat.2021.06.008 PMC8429620 34541411

[140] Hoemann, C. D. et al. Chitosan-glycerol phosphate/blood implants elicit hyaline cartilage repair integrated with porous subchondral bone in microdrilled rabbit defects. *Osteoarthr. Cartil.* **15**, 78–89 (2007). 10.1016/j.joca.2006.06.015 16895758

[141] Marchand, C., Rivard, G. E., Sun, J. & Hoemann, C. D. Solidification mechanisms of chitosan-glycerol phosphate/blood implant for articular cartilage repair. *Osteoarthr. Cartil.* **17**, 953–960 (2009). 10.1016/j.joca.2008.12.002 19152788

[142] Shen, E. C. et al. Releasing growth factors from activated human platelets after chitosan stimulation: a possible bio-material for platelet-rich plasma preparation. *Clin. Oral. Implants Res.* **17**, 572–578 (2006). 10.1111/j.1600-0501.2004.01241.x 16958699

[143] Deprés-Tremblay, G., Chevrier, A., Tran-Khanh, N., Nelea, M. & Buschmann, M. D. Chitosan inhibits platelet-mediated clot retraction, increases platelet-derived growth factor release, and increases residence time and bioactivity of platelet-rich plasma in vivo. *Biomed. Mater.* **13**, 015005 (2017). 10.1088/1748-605X/aa8469 29125132

[144] Dwivedi, G., Chevrier, A., Hoemann, C. D. & Buschmann, M. D. Injectable freeze-dried chitosan-platelet-rich-plasma implants improve marrow-stimulated cartilage repair in a chronic-defect rabbit model. *J. Tissue Eng. Regener. Med.* **13**, 599–611 (2019). 10.1002/term.2814 30706995

[145] Lafantaisie-Favreau, C.-H. et al. Subchondral pre-solidified chitosan/blood implants elicit reproducible early osteochondral wound-repair responses including neutrophil and stromal cell chemotaxis, bone resorption and repair, enhanced repair tissue integration and delayed matrix deposition. *BMC Musculoskelet. Disord.* **14**, 27 (2013). 10.1186/1471-2474-14-27 PMC3602124 23324433

[146] Kulkarni, M. et al. Wettability studies of topologically distinct titanium surfaces. *Colloids Surf. B-Biointerfaces* **129**, 47–53 (2015). 10.1016/j.colsurfb.2015.03.024 25819365

[147] Alfarsi, M. A., Hamlet, S. M. & Ivanovski, S. The effect of platelet proteins released in response to titanium implant surfaces on macrophage pro-inflammatory cytokine gene expression. *Clin. Implant Dent. Relat. Res.* **17**, 1036–1047 (2015). 10.1111/cid.12231 24909201

[148] Wu, S., Liu, X. & Gao, C. Role of adsorbed proteins on hydroxyapatite-coated titanium in osteoblast adhesion and osteogenic differentiation. *Sci. Bull.* **60**, 691–700 (2015).

[149] Tunali, M., Ozdemir, H., Kucukodaci, Z., Akman, S. & Firatli, E. In vivo evaluation of titanium-prepared platelet-rich fibrin (T-PRF): a new platelet concentrate. *Br. J. Oral. Maxillofac. Surg.* **51**, 438–443 (2013). 10.1016/j.bjoms.2012.08.003 22951383

[150] Cho, S.-A., Lee, B.-K., Park, S.-H. & Ahn, J.-J. The bone integration effects of platelet-rich fibrin by removal torque of titanium screw in rabbit tibia. *Platelets* **25**, 562–566 (2014). 10.3109/09537104.2013.856398 24433149

[151] Romero-Gavilan, F. et al. Protein adsorption/desorption dynamics on Ca-enriched titanium surfaces: biological implications. *J. Biol. Inorg. Chem.* **26**, 715–726 (2021). 10.1007/s00775-021-01886-4 PMC8437886 34453217

[152] Sabino, R. M., Kauk, K., Movafaghi, S., Kota, A. & Popat, K. C. Interaction of blood plasma proteins with superhemophobic titania nanotube surfaces. *Nanomed.-Nanotechnol. Biol. Med.* **21**, 102046 (2019). 10.1016/j.nano.2019.102046 PMC6814547 31279063

[153] Xu, R. et al. Zwitterionic PMCP-functionalized titanium surface resists protein adsorption, promotes cell adhesion, and enhances osteogenic activity. *Colloids Surf. B: Biointerfaces* **206**, 111928 (2021). 10.1016/j.colsurfb.2021.111928 34153618

[154] Rosales, A. M. & Anseth, K. S. The design of reversible hydrogels to capture extracellular matrix dynamics. *Nat. Rev. Mater.* **1**, 15012 (2016). 10.1038/natrevmats.2015.12 PMC5714327 29214058

[155] Tong, Z. et al. Adaptable hydrogel with reversible linkages for regenerative medicine: dynamic mechanical microenvironment for cells. *Bioact. Mater.* **6**, 1375–1387 (2021). 10.1016/j.bioactmat.2020.10.029 PMC7658331 33210030

[156] Yu, Z. et al. Application of fibrin-based hydrogels for nerve protection and regeneration after spinal cord injury. *J. Biol. Eng.* **14**, 22 (2020). 10.1186/s13036-020-00244-3 PMC7397605 32774454

[157] Lee, H. H. et al. Release of bioactive adeno-associated virus from fibrin scaffolds: effects of fibrin glue concentrations. *Tissue Eng. Part A* **17**, 1969–1978 (2011). 10.1089/ten.tea.2010.0586 PMC3142631 21449684

[158] Rocha, C. A. et al. Maxillary sinus lift response to platelet-rich plasma associated with autogenous bone, ceramic biphasic HA/beta-TCP (70:30), or deproteinized bovine bone. *Int. J. Implant Dent.* **6**, 79 (2020). 10.1186/s40729-020-00277-9 PMC7701205 33251558

[159] Son, S.-R. et al. Platelet-rich plasma encapsulation in hyaluronic acid/gelatin-BCP hydrogel for growth factor delivery in BCP sponge scaffold for bone regeneration. *J. Biomater. Appl.* **29**, 988–1002 (2015). 10.1177/0885328214551373 25234121

[160] Zhou, H. & Xu, H. H. K. The fast release of stem cells from alginate-fibrin microbeads in injectable scaffolds for bone tissue engineering. *Biomaterials* **32**, 7503–7513 (2011). 10.1016/j.biomaterials.2011.06.045 PMC3159408 21757229

[161] Vorwald, C. E., Gonzalez-Fernandez, T., Joshee, S., Sikorski, P. & Leach, J. K. Tunable fibrin-alginate interpenetrating network hydrogels to support cell spreading and network formation. *Acta Biomater.* **108**, 142–152 (2020). 10.1016/j.actbio.2020.03.014 PMC7198331 32173582

[162] Ho, W., Tawil, B., Dunn, J. C. & Wu, B. M. The behavior of human mesenchymal stem cells in 3D fibrin clots: dependence on fibrinogen concentration and clot structure. *Tissue Eng.* **12**, 1587–1595 (2006). 10.1089/ten.2006.12.1587 16846354

[163] Richter, A. et al. Triple modification of alginate hydrogels by fibrin blending, iron nanoparticle embedding, and serum protein-coating synergistically promotes strong endothelialization. *Adv. Mater. Interfaces* **8**, 2002015 (2021).

[164] Balgude, A. P., Yu, X., Szymanski, A. & Bellamkonda, R. V. Agarose gel stiffness determines rate of DRG neurite extension in 3D cultures. *Biomaterials* **22**, 1077–1084 (2001). 10.1016/s0142-9612(00)00350-1 11352088

[165] Yao, S. et al. Co-effects of matrix low elasticity and aligned topography on stem cell neurogenic differentiation and rapid neurite outgrowth. *Nanoscale* **8**, 10252–10265 (2016). 10.1039/c6nr01169a 27124547

[166] Du, J. et al. Prompt peripheral nerve regeneration induced by a hierarchically aligned fibrin nanofiber hydrogel. *Acta Biomater.* **55**, 296–309 (2017). 10.1016/j.actbio.2017.04.010 28412554

[167] Urist, M. R. Bone: formation by autoinduction. *Science* **150**, 893–899 (1965). 10.1126/science.150.3698.893 5319761

[168] van den Dolder, J., de Ruijter, A. J. E., Spauwen, P. H. M. & Jansen, J. A. Observations on the effect of BMP-2 on rat bone marrow cells cultured on titanium substrates of different roughness. *Biomaterials* **24**, 1853–1860 (2003). 10.1016/s0142-9612(02)00571-9 12615475

[169] Ao, Q. et al. Fibrin glue/fibronectin/heparin-based delivery system of Bmp2 induces osteogenesis in MC3T3-E1 cells and bone formation in rat calvarial critical-sized defects. *ACS Appl. Mater. Interfaces* **12**, 13400–13410 (2020). 10.1021/acsami.0c01371 32091872

[170] Fan, Q. et al. Implantable blood clot loaded with BMP-2 for regulation of osteoimmunology and enhancement of bone repair. *Bioact. Mater.* **6**, 4014–4026 (2021). 10.1016/j.bioactmat.2021.04.008 PMC8085758 33997490

[171] Berkmann, J. C. et al. In vivo validation of spray-dried mesoporous bioactive glass microspheres acting as prolonged local release systems for BMP-2 to support bone regeneration. *Pharmaceutics* **12**, 823 (2020). 10.3390/pharmaceutics12090823 PMC7559713 32872353

[172] Ren, W. H., Yang, L. J. & Dong, S. Z. Induction of reparative dentin formation in dogs with combined recombinant human bone morphogenetic protein 2 and fibrin sealant. *Chin. J. Dent. Res* **2**, 21–24 (1999). 10863412

[173] Liu, Y. et al. Segmental bone regeneration using an rhBMP-2-loaded gelatin/nanohydroxyapatite/fibrin scaffold in a rabbit model. *Biomaterials* **30**, 6276–6285 (2009). 10.1016/j.biomaterials.2009.08.003 19683811

[174] Ben-David, D. et al. Low dose BMP-2 treatment for bone repair using a PEGylated fibrinogen hydrogel matrix. *Biomaterials* **34**, 2902–2910 (2013). 10.1016/j.biomaterials.2013.01.035 23375953

[175] Huang, C. Y. C., Deitzer, M. A. & Cheung, H. S. Effects of fibrinolytic inhibitors on chondrogenesis of bone-marrow derived mesenchymal stem cells in fibrin gels. *Biomech. Model. Mechanobiol.* **6**, 5–11 (2007). 10.1007/s10237-006-0033-2 16691415

[176] Marcinczyk, M. et al. Laminin-111 enriched fibrin hydrogels for skeletal muscle regeneration. *Biomaterials* **141**, 233–2423 (2017). 10.1016/j.biomaterials.2017.07.003 28697464

[177] Park, K.-H., Kim, H., Moon, S. & Na, K. Bone morphogenic protein-2 (BMP-2) loaded nanoparticles mixed with human mesenchymal stem cell in fibrin hydrogel for bone tissue engineering. *J. Biosci. Bioeng.* **108**, 530–537 (2009). 10.1016/j.jbiosc.2009.05.021 19914589

[178] Dong Nyoung, H., Hospodiuk, M. & Ozbolat, I. T. Synergistic interplay between human MSCs and HUVECs in 3D spheroids laden in collagen/fibrin hydrogels for bone tissue engineering. *Acta Biomater.* **95**, 348–356 (2019). 10.1016/j.actbio.2019.02.046 30831326

[179] Carr, M. E. Jr, Shen, L. L. & Hermans, J. Mass–length ratio of fibrin fibers from gel permeation and light scattering. *Biopolymers* **16**, 1–15 (1977). 10.1002/bip.1977.360160102 14741

[180] Davis, H. E., Miller, S. L., Case, E. M. & Leach, J. K. Supplementation of fibrin gels with sodium chloride enhances physical properties and ensuing osteogenic response. *Acta Biomater.* **7**, 691–699 (2011). 10.1016/j.actbio.2010.09.007 20837168

[181] Hu, L. et al. Extracellular vesicles from bone marrow-derived macrophages enriched in ARG1 enhance microglial phagocytosis and haematoma clearance following intracerebral haemorrhage. *J. Extracell. Vesicles* **14**, e70041 (2025). 10.1002/jev2.70041 PMC11770371 39868438

[182] Zhang, G., Drinnan, C. T., Geuss, L. R. & Suggs, L. J. Vascular differentiation of bone marrow stem cells is directed by a tunable three-dimensional matrix. *Acta Biomater.* **6**, 3395–3403 (2010). 10.1016/j.actbio.2010.03.019 20302976

[183] Mendez, J. J. et al. Mesenchymal stromal cells form vascular tubes when placed in fibrin sealant and accelerate wound healing in vivo. *Biomaterials* **40**, 61–67 (2015). 10.1016/j.biomaterials.2014.11.011 PMC4268422 25433608

[184] Zhou, W. et al. Peptide-based inflammation-responsive implant coating sequentially regulates bone regeneration to enhance interfacial osseointegration. *Nat. Commun.* **16**, 3283 (2025). 10.1038/s41467-025-58444-8 PMC11973180 40189598

[185] Julier, Z., Park, A. J., Briquez, P. S. & Martino, M. M. Promoting tissue regeneration by modulating the immune system. *Acta Biomater.* **53**, 13–28 (2017). 10.1016/j.actbio.2017.01.056 28119112

[186] Tanaka, R., Saito, Y., Fujiwara, Y., Jo, J. -i & Tabata, Y. Preparation of fibrin hydrogels to promote the recruitment of anti-inflammatory macrophages. *Acta Biomater.* **89**, 152–165 (2019). 10.1016/j.actbio.2019.03.011 30862554

[187] Ohba, S. et al. Efficacy of platelet-rich plasma gel and hyaluronan hydrogel as carriers of electrically polarized hydroxyapatite microgranules for accelerating bone formation. *J. Biomed. Mater. Res. Part A* **100A**, 3167–3176 (2012). 10.1002/jbm.a.34250 22847859

[188] Zhao, Y. H. et al. The combined use of cell sheet fragments of periodontal ligament stem cells and platelet-rich fibrin granules for avulsed tooth reimplantation. *Biomaterials* **34**, 5506–5520 (2013). 10.1016/j.biomaterials.2013.03.079 23639531

[189] Dohan Ehrenfest, D. M., Rasmusson, L. & Albrektsson, T. Classification of platelet concentrates: from pure platelet-rich plasma (P-PRP) to leucocyte- and platelet-rich fibrin (L-PRF). *Trends Biotechnol.* **27**, 158–167 (2009). 10.1016/j.tibtech.2008.11.009 19187989

[190] Wu, C. L. et al. Platelet-rich fibrin increases cell attachment, proliferation and collagen-related protein expression of human osteoblasts. *Aust. Dent. J.* **57**, 207–212 (2012). 10.1111/j.1834-7819.2012.01686.x 22624763

[191] Song, Y. et al. Nano-biphasic calcium phosphate/polyvinyl alcohol composites with enhanced bioactivity for bone repair via low-temperature three-dimensional printing and loading with platelet-rich fibrin. *Int. J. Nanomed.* **13**, 505–523 (2018). 10.2147/IJN.S152105 PMC5790108 29416332

[192] Chen, F. M., Zhang, M. & Wu, Z. F. Toward delivery of multiple growth factors in tissue engineering. *Biomaterials* **31**, 6279–6308 (2010). 10.1016/j.biomaterials.2010.04.053 20493521

[193] Anitua, E., Sanchez, M. & Orive, G. Potential of endogenous regenerative technology for in situ regenerative medicine. *Adv. Drug Deliv. Rev.* **62**, 741–752 (2010). 10.1016/j.addr.2010.01.001 20102730

[194] Jooybar, E. et al. Developing hyaluronic acid microgels for sustained delivery of platelet lysate for tissue engineering applications. *Int. J. Biol. Macromol.* **144**, 837–846 (2020). 10.1016/j.ijbiomac.2019.10.036 31715235

[195] Santo, V. E., Gomes, M. E., Mano, J. F. & Reis, R. L. Chitosan-chondroitin sulphate nanoparticles for controlled delivery of platelet lysates in bone regenerative medicine. *J. Tissue Eng. Regen. Med.* **6**, s47–s59 (2012). 10.1002/term.1519 22684916

[196] Sadeghinia, A., Davaran, S., Salehi, R. & Jamalpoor, Z. Nano-hydroxy apatite/chitosan/gelatin scaffolds enriched by a combination of platelet-rich plasma and fibrin glue enhance proliferation and differentiation of seeded human dental pulp stem cells. *Biomed. Pharmacother.* **109**, 1924–1931 (2019). 10.1016/j.biopha.2018.11.072 30551447

[197] Siddiqui, J. A. & Partridge, N. C. Physiological bone remodeling: systemic regulation and growth factor involvement. *Physiology* **31**, 233–245 (2016). 10.1152/physiol.00061.2014 PMC6734079 27053737

[198] Kiernan, C.H., Wolvius, E.B., Brama, P.A.J., Farrell, E. The immune response to allogeneic differentiated mesenchymal stem cells in the context of bone tissue engineering. *Tissue Eng. Part B Rev.* **24**, 75–83 (2018). 10.1089/ten.TEB.2017.0175 28816104

[199] Wu, M., Chen, G. & Li, Y. P. TGF-beta and BMP signaling in osteoblast, skeletal development, and bone formation, homeostasis and disease. *Bone Res.* **4**, 16009 (2016). 10.1038/boneres.2016.9 PMC4985055 27563484

